# Integriertes Versorgungsmanagement für chronisch erkrankte ältere Menschen in der eigenen Häuslichkeit: Evidenz aus Cochrane-Reviews

**DOI:** 10.1007/s00391-020-01796-1

**Published:** 2020-10-12

**Authors:** Anne Göhner, Eva Maria Bitzer, Elena Dreher, Erik Farin-Glattacker, Bernhard Heimbach, Katharina Kohler, Andy Maun, Gloria Metzner, Sebastian Voigt-Radloff

**Affiliations:** 1 Zentrum für Geriatrie und Gerontologie Freiburg, Universitätsklinikum Freiburg, Medizinische Fakultät, Albert-Ludwigs-Universität Freiburg, Lehener Straße 88, 79106 Freiburg, Deutschland; 2 Public Health & Health Education, Pädagogische Hochschule Freiburg, Freiburg, Deutschland; 3 Sektion Versorgungsforschung und Rehabilitationsforschung, Universitätsklinikum Freiburg, Medizinische Fakultät, Albert-Ludwigs-Universität Freiburg, Freiburg, Deutschland; 4 Institut für Allgemeinmedizin, Universitätsklinikum Freiburg, Medizinische Fakultät, Albert-Ludwigs-Universität Freiburg, Freiburg, Deutschland; 5 Institut für Evidenz in der Medizin (für Cochrane Deutschland Stiftung), Universitätsklinikum Freiburg, Medizinische Fakultät, Albert-Ludwigs-Universität Freiburg, Freiburg, Deutschland

**Keywords:** Hochaltrig, Multimorbid, Gesundheit, Koordiniert, Schnittstelle, Aged, Multimorbid, Health Care, Coordinated Care, Community

## Abstract

**Hintergrund:**

Die Anzahl multipel chronisch erkrankter Älterer steigt, und Multimorbidität geht mit hoher Inanspruchnahme von Gesundheitsleistungen einher. Um Selbstständigkeit und Verbleib in der Häuslichkeit zu erhalten, wird zunehmend ein integriertes Versorgungsmanagement eingesetzt. Zur Wirksamkeit in der Zielgruppe der multipel chronisch erkrankten Älteren liegen aber kaum belastbare Daten vor.

**Ziel der Arbeit:**

Bewertung der Wirksamkeit von integriertem Versorgungsmanagement bei Erwachsenen und Abschätzung der Übertragbarkeit auf ältere, multimorbide Personen in Deutschland.

**Methoden:**

Systematische Literaturrecherche in der *Cochrane Library* mit Einschluss von Cochrane-Reviews (CR) zu (a) den 13 häufigsten Gesundheitsproblemen im Alter, mit (b) Komponenten des integrierten Versorgungsmanagements bei (c) Erwachsenen jeden Alters. Experten schätzten die Übertragbarkeit der eingeschlossenen CR auf multipel chronisch erkrankte Ältere in Deutschland ein.

**Ergebnisse:**

Aus 1412 Treffern wurden 126 CR eingeschlossen. Zur Endpunktkategorie Selbstständigkeit und funktionale Gesundheit zeigten 25 CR klinisch relevante Ergebnisse mit moderater Evidenzqualität. Folgende Interventionskomponenten wurden – unter Berücksichtigung identifizierter Barrieren – als übertragbar eingeschätzt und könnten für ein effektives, indikationsspezifisch integriertes Versorgungsmanagement multipel chronisch erkrankter Älterer herangezogen werden: (1) körperliche Aktivierung, (2) multidisziplinäre Interventionen, (3) das Selbstmanagement verstärkende Interventionen, (4) kognitive Therapieverfahren, (5) telemedizinische Interventionen und (6) Disease-Management-Programme.

**Schlussfolgerungen:**

Die identifizierten Komponenten sollten in versorgungs- und patientennahen randomisierten kontrollierten Studien auf Wirksamkeit bei gebrechlichen Älteren geprüft werden.

**Zusatzmaterial online:**

Zusätzliche Informationen sind in der Online-Version dieses Artikels (10.1007/s00391-020-01796-1) enthalten.

## Abstract (en)

**Background:**

The number of multiple chronically ill older people is increasing and multimorbidity is associated with high utilization of health services. Integrated care management is increasingly used to address this problem; however, there is a substantial lack of reliable data on its effectiveness in this target group.

**Objective:**

To assess the effectiveness of components of integrated care management in adults of all ages and to estimate the transferability to older, multimorbid people in Germany.

**Methods:**

A systematic search was carried out in the Cochrane Library for Cochrane reviews (CR) on (a) the 13 most frequent health problems in old age, which (b) evaluated components of integrated care management in (c) adults of all ages. Experts assessed the transferability of the included CR to multiple chronically ill older people in Germany.

**Results:**

Out of 1412 hits 126 CR were included. Regarding independence and functional health outcomes, 25 CR showed clinically relevant results with at least a moderate level of evidence. The following intervention components were estimated to be transferable and could be adapted to be part of an effective integrated care management for multimorbid chronically ill older people, specified by indications and taking identified barriers into account: (1) physical activation, (2) multidisciplinary interventions (3) interventions that enhance self-management, (4) cognitive therapy modalities, (5) telemedical interventions and (6) disease management programs.

**Conclusion:**

The effectiveness of the identified components in frail older patients should be assessed in care-related and patient-related randomized controlled studies.

## Kurze Hinführung zum Thema

Integrierte Versorgung ist dadurch gekennzeichnet, dass Gesundheitsleistungen koordiniert nach den Bedarfen der betroffenen Personen ausgeführt werden und kontinuierlich an den Versorgungsübergängen zur Verfügung stehen [6, 41, 48, 49]. Zwar sind deren Komponenten zunehmend klarer benannt [8, 19]. Zur Wirksamkeit bei multipel chronisch erkrankten Älteren liegen aber nahezu keine belastbaren Daten vor [51]. Diese Arbeit präsentiert wirksame Komponenten integrierter Versorgung und deren Übertragbarkeit auf die Zielgruppe in Deutschland.

## Hintergrund

Laut Gesundheitsberichtserstattung des Bundes von 2015 [32] sind die häufigsten Erkrankungen und Gesundheitsprobleme von Menschen über 65 Jahren in Deutschland (1) Hypertonie (>50 %) und (2) weitere kardiovaskuläre Erkrankungen, inkl. Schlaganfall (>35 %), (3) Folgen von Polypharmazie (>30 %), (4) Arthrose (>29 %), (5) chronische Rückenschmerzen (>24 %), (6) Inkontinenz (>23 %), (7) Diabetes mellitus (>19 %), (8) Depression (>17 %), (9) Krebserkrankungen (>16 %), (10) chronische Atemwegserkrankungen (>15 %), (11) Folgen von Verletzungen oder Stürzen (>10 %), (12) schwere visuelle Einschränkungen (>10 %) und (13) Demenz (>6 %). Die Anzahl multipel chronisch erkrankter älterer Menschen steigt [20], und Multimorbidität geht mit hoher Inanspruchnahme von Gesundheitsleistungen einher [26]. Für die komplexe Behandlung multipel chronisch erkrankter älterer Menschen wird zunehmend ein integriertes Versorgungsmanagement eingesetzt [45].

Zur Wirksamkeit integrierter Versorgung bei älteren Patienten i. Allg. liegen nur wenige und für multipel chronisch erkrankte Ältere nahezu keine belastbaren Daten vor [51]. Bei massivem Evidenzmangel für diese wachsende Zielgruppe kann neben klinischer Erfahrung auch das Heranziehen von Studien mit jüngeren, nicht multipel erkrankten Patienten sinnvoll sein [31, 42]. Jedoch sollte die Übertragbarkeit gewissenhaft geprüft werden [33]. Daher untersucht die vorliegende Übersichtsarbeit Cochrane-Reviews (CR) zu Interventionen, die (a) Komponenten des integrierten Versorgungsmanagements in (b) erwachsenen Populationen evaluieren und gleichzeitig (c) Erkrankungen adressieren, die bei älteren Menschen häufig sind. So werden zwei Ziele verfolgt:

1. Wirksamkeit von Komponenten des integrierten Versorgungsmanagements untersuchen und bewerten. Im Fokus stehen erwachsene Populationen mit mindestens einer chronischen Erkrankung sowie die Wirksamkeit der Komponenten auf (1) Selbstständigkeit und funktionale Gesundheit im Alltag, (2) Symptomreduktion, (3) Inanspruchnahme von Gesundheitsleistungen, (4) Mortalität und (5) unerwünschte Ereignisse bei dieser Zielgruppe.
2. Deren Übertragbarkeit auf ältere, multimorbide Populationen in Deutschland abschätzen.

Bei der Übertragbarkeit der Interventionen und Effekte sind nicht Alter und Multimorbidität per se als kritische Aspekte zu berücksichtigen, sondern viel mehr die häufig damit einhergehende eingeschränkte Funktionalität, die wiederum eng mit dem Konzept der Frailty (Gebrechlichkeit) verbunden ist [7].

## Methoden

Es erfolgte eine systematische Literaturrecherche nach Cochrane Reviews (CRs) in der *Cochrane Library* via *PubMed* bis zum 31.07.2019. Die Übertragbarkeit der Interventionskomponenten wurde durch 2 ExpertInnen in geriatrischer Versorgung und sozialer Gerontologie anhand folgender Dimensionen geprüft: (1) „Primärstudien mit Älteren liegen vor“, (2) „Intervention ist in kontinuierliche Versorgung integrierbar“, (3) „Intervention ist in komplexe Programme integrierbar“ und (4) „Intervention wurde bereits im deutschen Versorgungssetting untersucht oder implementiert oder ist mit in Deutschland qualifiziertem Personal umsetzbar“. Die Prüfergebnisse wurden mit 5 weiteren ExpertInnen aus den Bereichen Allgemeinmedizin, Geriatrie, Psychologie, Versorgungsforschung und Gesundheitswissenschaft beraten. Ein- und Ausschlusskriterien wurden vorab definiert (Zusatzmaterial online: Supplement 1). Funktionseinschränkungen können Interventionseffekte einschränken, deshalb wurden nur CR eingeschlossen, die in Metaanalysen mit mindestens 2 Primärstudien signifikante und stabile, moderate Effekte gegenüber einer Kontrollbedingung nachwiesen. Ältere Patienten mit Funktionseinschränkungen oder Gebrechlichkeit können meist nur wenige prioritäre Behandlungsziele verfolgen. Gleichzeitig präferiert diese Patientengruppe in der Regel ein möglichst langes und selbstbestimmtes Leben in der eigenen Häuslichkeit [12]. Daher fokussiert die Übersicht die Wirksamkeit der Interventionen auf Endpunkte der Bereiche „Selbstständigkeit und funktionale Gesundheit im Alltag“. Die Reihenfolge der Erkrankungen in der Ergebnisdarstellung folgt den Häufigkeiten der Grunderkrankungen in der Bevölkerung [48]. Weitere Informationen zur methodischen Vorgehensweise finden sich im Zusatzmaterial online: Supplement 1.

## Ergebnisse

**Figure 1:**
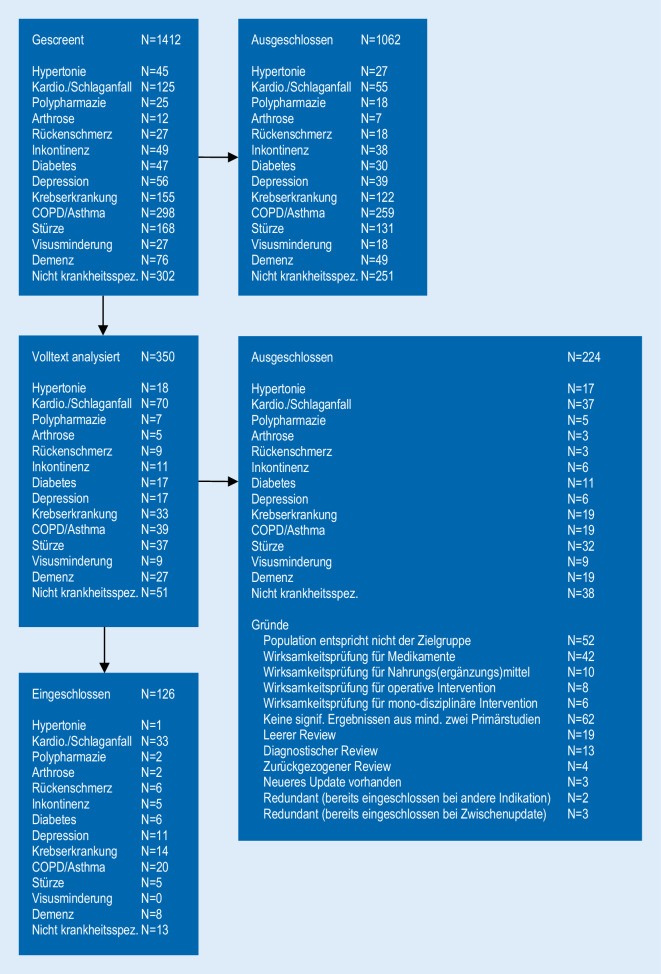

Aus insgesamt 1412 Treffern wurden 350 Volltexte analysiert und 126 Cochrane Reviews (CR) final eingeschlossen (Abb. 1). Eine Übersicht über die Ergebnisse aller eingeschlossenen CR findet sich im Zusatzmaterial online: Supplement 2. Insgesamt 25 CR zeigen klinisch relevante Ergebnisse mit mindestens moderater Evidenzqualität für Endpunkte in den Bereichen Selbstständigkeit und funktionale Gesundheit, davon 7 zum Schlaganfall [10, 11, 24, 25, 28, 29, 40], je 4 zu Rückenschmerzen [18, 23, 34, 44] und Atemwegserkrankungen (Asthma, Chronisch obstruktive Lungenerkrankung [COPD]) [14, 21, 27, 50], 3 zur Demenz [1, 22, 47] und 2 zu Stürzen [13, 37]. Jeweils ein CR fand sich zu Arthrose [16], Diabetes mellitus [3], Depression [17], kardiologischen Erkrankungen [38] und chronischen Gesundheitsproblemen ([9]; Tab. 1). Zu Bluthochdruck, Inkontinenz, Krebserkrankung, Polypharmazie und schweren visuellen Einschränkungen konnten keine CR mit mindestens moderater Evidenzqualität und klinisch bedeutsamen Effekten auf Selbstständigkeit und funktionale Gesundheit identifiziert werden.

**Table 1:** 

Erstautor, Jahr	Interventionskomponente mit nachgewiesener Wirksamkeit auf Selbstständigkeit und funktionale Gesundheit	Anwendungsoption für Ältere in Deutschland				
*Kardiovaskuläre Erkrankungen, inkl. Schlaganfall*		*65+*	*KV*	*KP*	*D*	*B*
Sibilitz 2016 [38]	Rehabilitationstraining nach Herzklappenoperation	–	+	+	+	B1
*Schlaganfall*						
English 2017[10]	Zirkeltraining	+	+	+	+	B1
French 2016 [11]	Repetitives Aufgabentraining der unteren Extremitäten	+	+	+	+	B1
Mehrholz 2017[25]	Elektromechanisch und robotisch unterstütztes Gangtraining plus Physiotherapie	+	–	+	+	B2
Mehrholz 2018 [24]	Elektromechanisch und robotisch unterstütztes Armtraining	+	–	+	+	B2
Pollock 2014a [29]	Spezifisches Aufstehtraining	–	+	+	+	B1
Pollock 2014b [28]	Physikalische Rehabilitation	–	+	+	+	B1
Thieme 2018 [40]	Spiegeltherapie	+	+	+	+	
*Arthrose*		*65+*	*KV*	*KP*	*D*	*B*
Hurley 2018 [16]	Mehrere Komponenten umfassendes, körperliches Training bei Hüft- oder Kniearthrose	+	+	+	+	B1
*Rückenschmerz*		*65+*	*KV*	*KP*	*D*	*B*
Kamper 2014 [18]	Multidisziplinäre biopsychosoziale Rehabilitation (chronisch)	–	+	+	+	B1
Marin 2017 [23]	Multidisziplinäre biopsychosoziale Rehabilitation (subakut)	–	+	+	+	B1
Saragiotto 2016 [34]	Übungen zur Bewegungskontrolle (chronisch)	+	+	+	+	B1
Wieland 2017 [44]	Yoga (chronisch)	–	+	+	–	B1
*Diabetes mellitus*		*65+*	*KV*	*KP*	*D*	*B*
Baumeister 2012 [3]	Psychologische Interventionen gegen Depression	+	+	+	+	B3
*Depression*		*65+*	*KV*	*KP*	*D*	*B*
Ijaz 2018 [17]	Psychotherapie	+	+	+	+	B3
*Chronisch obstruktive Lungenerkrankung (COPD)/Asthma*		*65+*	*KV*	*KP*	*D*	*B*
Howcroft 2016 [14]	Aktionsplan mit Patientenschulung (COPD)	+	+	+	+	B3
Kruis 2013 [21]	Integrierte Disease-Management-Programme (COPD)	+	+	+	+	B4
Peytremann 2015 [27]	Spezielle Disease-Management-Programme (Asthma)	+	+	+	+	B4
Yang 2016 [50]	Yoga (Asthma)	–	+	+	–	B1
*Stürze*		*65+*	*KV*	*KP*	*D*	*B*
Hopewell 2018 [13]	Multifaktorielle und Multikomponenteninterventionen	+	+	+	+	–
Sherrington 2019 [37]	Übungen zur Vorbeugung von Stürzen	+	+	+	+	B1
*Demenz*		*65+*	*KV*	*KP*	*D*	*B*
Bahar-Fuchs 2019 [1]	Kognitives Training	+	+	+	+	–
Lins 2014 [22]	Telefonberatung für Angehörige	+	+	+	+	–
Woods 2018 [47]	Reminiszenztherapie	+	+	+	+	–
*Nicht krankheitsspezifische Cochrane Reviews*		*65+*	*KV*	*KP*	*D*	*B*
Coulter 2015 [9]	Personalisierte Zielvereinbarung und Aktionsplan	+	+	+	+	–

Titelleiste: *65+* Primärstudien mit Älteren liegen vor. *KV* Intervention integrierbar in kontinuierliche Versorgung. *KP* Intervention integrierbar in komplexe Programme. *D* Intervention wurde bereits im deutschen Versorgungskontext untersucht oder implementiert oder ist mit in Deutschland qualifiziertem Personal umsetzbar. *B* Barrieren, die bei der Übertragung in die integrierte Versorgung Älterer zu berücksichtigen sind. *B1* Bei körperlich intensiven Trainingsverfahren sind reduzierte Belastbarkeit sowie Sturz- und Verletzungsgefahr Älterer besonders zu beachten. *B2* Teure und installierungsaufwendige Geräte erschweren die Anschaffung und damit die Kontinuität zwischen Versorgungsformen. *B3* Kognitive Einschränkungen Älterer können mental anspruchsvolle Interventionen erschweren. *B4* Krankheits- und kassenspezifische Einschreibungsmodalitäten erschweren die Integration in komplexe Programme für multimorbide Ältere

## Diskussion

Folgende Interventionskomponenten wurden als übertragbar eingeschätzt und könnten für ein effektives integriertes Versorgungsmanagement multipel chronisch erkrankter Älterer in Deutschland herangezogen werden:

1. Durch Schulung und qualifiziertes Training vermittelte körperliche Aktivierung bei Schlaganfall, Herzklappenoperation, Arthrose, Rückenschmerzen und Stürzen. Wenn Trainingsverfahren vorwiegend bei jüngerer Klientel untersucht wurden [29, 38, 50], sind bei einer Übertragung auf ältere Patienten deren reduzierte Belastbarkeit sowie Sturz- und Verletzungsgefahr zu beachten. Ebenso könnte der Einsatz teurer Trainingsgeräte [24, 25] die Versorgungskontinuität erschweren, z. B. am Übergang von ressourcenstarken Maximalversorgern im städtischen Bereich zu ressourcenschwächeren Einzelpraxen in ländlichen Regionen. Die Durchführungsqualität von Yoga-Übungen [44, 50] könnte durch eine nichtstandardisierte Qualifizierung im deutschen Versorgungskontext gemindert werden.
2. Multidisziplinäre Interventionen bei Schlaganfall, Rückenschmerz und Stürzen. Die Rückenschmerzstudien [18, 23] und die Studie zu Schlaganfall [28] sind vorwiegend mit jüngerer Klientel durchgeführt worden. Körperliches Training und Patientenedukation scheinen für ältere Patienten jedoch ebenso durchführbar.
3. Das Selbstmanagement verstärkende Interventionen bei chronischen Atemwegserkrankungen, Diabetes mellitus, Depression und weiteren multipel, chronischen Erkrankungen [3, 9, 14, 17]. Als Barrieren könnten kognitive Einschränkungen wirken [2, 35].
4. Kognitive und psychosoziale Therapieverfahren bei Demenz [1, 47],
5. Telemedizinische Interventionen bei versorgenden Angehörigen mit hoher zeitlicher Belastung oder Patienten im ländlichen Raum mit weiten Zugangswegen [22] und
6. Disease-Management-Programme bei chronischen Atemwegserkrankungen [21, 27], wenn der Einschreibemodus kostenträgerübergreifend multimorbide Patienten zulässt.

Aufgrund des bestehenden Evidenzmangels in der Population der multipel chronisch erkrankten Älteren erweiterten wir die Zielgruppe unserer Übersichtsarbeit auf Erwachsene jeden Alters. Dadurch ist die direkte Übertragbarkeit limitiert. Eventuelle Implementierungsschwierigkeiten und möglicherweise geringere Effekte sind daher bei den jeweiligen Therapieentscheidungen individuell zu berücksichtigen. Eine weitere Limitation ist die Fokussierung auf die häufigsten Gesundheitsprobleme im Alter, ohne dass in den Primärstudien Gebrechlichkeit als geriatrietypisches Merkmal systematisch erfasst wurde. Zudem könnten die Begrenzung auf CR und der Verzicht auf die methodische Qualitätsbeurteilung der eingeschlossenen Reviews dazu geführt haben, dass Effekte nicht berücksichtigt oder überbewertet wurden. Die Ergebnisse aus anderen Übersichtsarbeiten und neueren Primärstudien zur integrierten Versorgung zeigen indes ein ähnliches Bild wie unsere Ergebnisse [4, 5, 15, 26, 30, 36, 43, 46]. Die identifizierten Komponenten sollten in weiteren Studien auf ihre Wirksamkeit bei gebrechlichen älteren Menschen in Deutschland überprüft werden. Um den prädiktiven Zusammenhang zwischen Gebrechlichkeit und Therapieeffekten ermitteln zu können, sollte dabei der Grad der Gebrechlichkeit zur Baseline mit harmonisierten Erfassungsinstrumenten erhoben werden.

Es besteht Bedarf an versorgungsnahen Studien, die in randomisiertem kontrolliertem Design patientenorientierte Endpunkte evaluieren. Beleuchtet werden sollten insbesondere Selbstständigkeit, funktionale Gesundheit, unerwünschte Ereignisse und die Langzeitwirkung der Interventionen. Da integrierte Versorgung meist ressourcenaufwendig ist, sollten Risikoscreenings und Eingangsassessments eine hohe Indikationsqualität sichern, d. h., diejenigen Patienten identifizieren, für die eine komplexe Behandlung indiziert und ein Therapieerfolg wahrscheinlich ist. Ein neueres Konstrukt für die Erfolgsmessung könnte die sog. Lebensraummobilität sein, die körperliche Funktionseinschränkungen und soziale Isolation in einem Indikator repräsentiert. Lebensraummobilität kann durch Sensortechnik untermauert werden und ist assoziiert mit körperlicher und mentaler Funktionsfähigkeit, Stürzen, Mortalität, Inanspruchnahmeverhalten sowie Aufnahmen in Langzeitpflegeeinrichtungen [39].

## Fazit für die Praxis

- Bei der integrierten Versorgung werden Gesundheitsleistungen koordiniert nach den Bedarfen der betroffenen Personen ausgeführt und stehen kontinuierlich und ohne Brüche an den Versorgungsübergängen zur Verfügung.
- Zur Wirksamkeit integrierter Versorgung für multipel chronisch erkrankte Ältere liegen nahezu keine belastbaren Daten vor.
- An Erwachsenen jeden Alters erprobte und indikationsspezifisch auf multipel chronisch erkrankte Ältere in Deutschland übertragbare Interventionskomponenten sind: körperliche Aktivierung, multidisziplinäre Interventionen, das Selbstmanagement verstärkende Interventionen, kognitive Therapieverfahren, telemedizinische Interventionen und Disease-Management.
- Bei der Übertragung sind identifizierte Besonderheiten und Barrieren zu berücksichtigen.
- Die Komponenten sollten in randomisierten kontrollierten, versorgungs- und patientennahen Studien auf Wirksamkeit bei gebrechlichen Älteren geprüft werden.

## Caption Electronic Supplementary Material




