# Supplementary material for: Integrated care management for older people with chronic diseases in domesticity: evidence from Cochrane reviews
Source: Z Gerontol Geriatr. 2020 Oct 12;54(1):54–60. [Article in German] doi: 10.1007/s00391-020-01796-1 (PMC7835300; doi:10.1007/s00391-020-01796-1)
Supplement: Supplementary file 1 [file 391_2020_1796_MOESM1_ESM.docx]

**Supplement 1: Details zur methodischen Vorgehensweise**

*Suche:* Alle CRs zu erwachsenen Personen bis zum 31.07.2019 wurden in der Pubmed Datenbank über die Filter *“The Cochrane database of systematic reviews”[Journal]* und *„Adult: 19+ years“* identifiziert. Für die häufigsten Gesundheitsprobleme im Alter wurden Synonyme kombiniert und als AND-Verknüpfung mit der CR Suche verbunden.

**Tabelle 1: Suchstrategie in pubmed**

| **Thema** | **Suche** | **Treffer** |
| --- | --- | --- |
| Begrenzung | Filter: Publication date to 2019/07/31; Adult: 19+ years |  |
|  | (“The Cochrane database of systematic reviews”[Journal]) AND… |  |
| Hypertension | (Hypertensi*[Title] OR (blood[Title] AND pressure[Title]) OR (systolic[Title] AND pressure[Title]) OR (diastolic[Title] AND pressure[Title])) | 45 |
| Cardio-vascular | (heart[Title] OR stroke[Title] OR cardi*[Title]) | 125 |
| Polypharmacy | ((drug*[Title] OR medic*[Title]) AND review*[Title]) OR medication[Title] OR prescri* [Title] OR polypharmac*[Title]) | 25 |
| Osteoarthritis | ((degenerati*[Title] AND arthritis[Title]) OR Osteoarthrit*[Title]) | 12 |
| Back pain | (back[Title] AND pain[Title]) | 27 |
| Incontinence | incontinen*[Title] | 49 |
| Diabetes | (insulin[Title] OR diabet*[Title]) | 47 |
| Depression | depress*[Title] | 56 |
| Cancer | (cancer[Title] OR neoplas*[Title]) | 155 |
| COPD | (COPD[Title] OR obstructive[Title] OR Asthma[Title] OR bronch*[Title]) | 298 |
| Falls | (fall*[Title] OR injur*[Title] OR acciden*[Title] OR trauma*[Title] OR (fractur*[Title] AND hip[Title]) OR (fractur*[Title] AND femoral[Title])) | 168 |
| Visus | (visu*[Title] OR macular*[Title]) | 27 |
| Dementia | (dement*[Title] OR Alzheimer[Title]) | 76 |
| Care | (care[Title] OR collab*[Title] OR manag*[Title] OR tele*[Title] OR step*[Title] OR integrat*[Title] OR plan*[Title] OR centered[Title] OR centred[Title] OR *sector*[Title] OR self*[Title] OR media[Title] OR internet[Title] OR computer[Title] OR discharg*[Title]) | 302 |

*Ein-/Ausschluss und Qualitätsbewertung:* Ein- und Ausschlusskriterien wurden durch das PICOS-Schema präzisiert und finden sich in Tabelle 2. Auf eine Bewertung der Reviewqualität wurde aus pragmatischen Gründen verzichtet, da CRs sowohl bei Protokollerstellung als auch bei finaler Publikation im Peer-Review Verfahren begutachtet werden.

**Tabelle 2: Ein- und Ausschlusskriterien**

|  | **Einschluss** | **Ausschluss** |
| --- | --- | --- |
| P | - Erwachsene Personen (19+) in der eigenen Häuslichkeit oder am Übergang dazu (z.B. Entlassmanagement) und mit mindestens einem der folgenden Gesundheitsprobleme: Bluthochdruck, kardiovaskuläre Erkrankung inkl. Schlaganfall, Polypharmazie, Arthrose, chronische Rückenschmerzen, Inkontinenz, Diabetes Mellitus, Depression, Krebserkrankung, Atemwegserkrankung, Stürze, schwere visuelle Einschränkung, Demenz. | - Jüngere Personen oder Menschen im Pflegeheim oder Akutkrankenhaus - Personen ohne die aufgeführten Gesundheitsprobleme |
| I | - Zwei oder mehr medizinische Disziplinen sind an der Intervention beteiligt bzw. werden in Kooperation oder Kommunikation geschult. - Telemedizinische Interventionen über Telefon, Internet oder spezifische Kommunikationssysteme. - Case Management oder ähnliche Interventionen, die Koordination, individuelle Planung oder Vermittlung von Versorgungsleistungen umfassen. - Aufbau oder Stärkung des sozialen Netzes inkl. Angehörigenberatung und -unterstützung. - Patientenschulung und -unterstützung zur Stärkung des Selbstmanagement inkl. adäquater Nutzung von sozialen und medizinischen Versorgungsangeboten. - Folgende Intervention, wenn sie in ein interdisziplinäres Versorgungsmanagement oder eine Stärkung des Patientenselbstmanagement integriert werden können.   - Psychotherapeutische, psychosoziale oder trainierende Verfahren durch nur eine Berufsgruppe   - Psychotherapeutische oder psychosoziale Interventionen bei somatischen Erkrankungen.   - Interventionen, die sich für die „Eigenanwendung“ durch geschulte Patienten eignen; z.B. körperliches Training, kognitiv-behaviorale Interventionen zur Lebenstil- oder Ernährungsumstellungen,.   - Interventionen zum Medikationsmanagement inkl. Zielwert-Studien z.B. bei Blutdruck oder Blutzucker | - Untersuchungen zu Effekten von Medikamenten oder Operationsmethoden - Untersuchungen zu Effekten von Nahrungs- oder Nahrungsergänzungsmitteln |
| C | Keine Spezifizierung |  |
| O | - Keine Spezifizierung der Endpunkte. Sie werden aber für die Ergebnisdarstellung patientenorientiert gruppiert. - Endpunkte, auf denen sich in einer Metaanalyse mit mindestens zwei Primärstudien signifikante Effekte zeigen | - CRs ohne Primärstudien (leere Reviews) - CRs ohne Metaanalysen - CRs, die ausschließlich Metaanalysen mit nur einer Primärstudie oder ohne signifikante Effekte enthalten. |
| S | - CRs zu Effekten von Interventionen - CRs zu Effekten einer Diagnostik- oder Screening-Nutzung - Das jeweils neuste Update des CRs | - CR Protokolle - Reine Diagnostik-CRs - CRs mit neuerem Update - CRs die zurückgezogen wurden - CRs für die das Update zurückgezogen wurde |

P=Population, I=Intervention, C=Comparison (Vergleichsgruppe), O=Outcome (Endpunkt), S=Studiendesign

*Datenextraktion und Ergebnisdarstellung:* Die Extraktion erfolgte für Endpunkte, für die aus einer Metaanalyse mit mindestens zwei Primärstudien signifikante Unterschiede zwischen Intervention- und Vergleichsgruppe berichtet wurden. Extrahiert wurden die verglichenen Interventionen sowie die entsprechenden Endpunkte mit den Werten der Effektschätzer inkl. Konfidenzintervall und der Evidenzqualität. Die extrahierten Endpunkte wurden in indikationsspezifischen Ergebnistabellen unter sechs Endpunktkategorien zusammengeführt: (1) Selbständigkeit und funktionale Gesundheit im Alltag, (2) Symptomreduktion, (3) Inanspruchnahme und gesundheitsökonomische Endpunkte, (4) Mortalität, (5) unerwünschte Ereignisse und (6) sonstige.

*Übertragbarkeit:* Barrieren zur Übertragbarkeit wurden nur für Interventionen geprüft, die in der Endpunktkategorie (1) Selbständigkeit und funktionale Gesundheit im Alltag klinisch relevante Effekte mit ausgewiesener und mindestens moderater Evidenzqualität aufwiesen. Denn Selbständigkeit und funktionale Gesundheit im Alltag sind für ältere Patienten die prioritären Ziele therapeutischer Maßnahmen [1-3]. Überprüft wurden potentielle Barrieren für eine kontinuierliche Versorgung, die auch an den Schnittstellen stabil bleiben muss, also z.B. an den Versorgungsübergängen von stationär zu ambulant, von städtischen zu ländlichen Regionen oder von ressourcenstarken Maximalversorgern zur kleineren Versorgungseinrichtungen. Ebenso wurde untersucht, ob die identifizierten Interventionen bereits in Deutschland durchgeführt oder international in Samples von älteren Menschen wissenschaftlich untersucht wurden und ob sie von in Deutschland qualifiziertem Gesundheitspersonal durchgeführt werden könnten [4].

1. Akishita M, Ishii S, Kojima T, Kozaki K, Kuzuya M, Arai H, Arai H, Eto M, Takahashi R, Endo H, Horie S, Ezawa K, Kawai S, Takehisa Y, Mikami H, Takegawa S, Morita A, Kamata M, Ouchi Y, Toba K: Priorities of health care outcomes for the elderly. J Am Med Dir Assoc 2013 Jul; 14(7): 479-484.
2. Fried TR, Tinetti M, Agostini J, Iannone L, Towle V: Health outcome prioritization to elicit preferences of older persons with multiple health conditions. Patient Educ Couns 2011 May; 83(2): 278-282.
3. Junius-Walker U, Wiese B, Klaaßen-Mielke R, Theile G, Müller CA, Hummers-Pradier E: Older patients' perceived burdens of their health problems: a cross-sectional analysis in 74 German general practices. Patient Prefer Adherence 2015 Jun 18; 9: 811-820.
4. Müller C, Glässel A, Marotzki U, Voigt-Radloff-S: Potenzialanalyse zu ergotherapeutischem Alltagstraining nach Schlaganfall. Z Evid Fortbild Qual Gesundhwesen 2014; 108(1): 36-44.
