# Supplementary material for: Integrated care management for older people with chronic diseases in domesticity: evidence from Cochrane reviews
Source: Z Gerontol Geriatr. 2020 Oct 12;54(1):54–60. [Article in German] doi: 10.1007/s00391-020-01796-1 (PMC7835300; doi:10.1007/s00391-020-01796-1)
Supplement: Supplementary file 2 [file 391_2020_1796_MOESM2_ESM.docx]

**Supplement 2**

**Evidence from Cochrane Reviews for Care Management Interventions for People with Chronic Diseases**

**Legend:**

**Black Letters:** Effects in favor of Intervention Group**; Red Letters:** Effects in favor of Control Group

**6mWD**: six-minute walk distance (meters); **6mWT:** six-minute Walking Test (min), **ADL**: Activities of daily living; **AQLQ**: Asthma Quality of Life Questionnaire; **BBS**: Berg Balance Scale; **BDI**: Beck Depression Inventory; **BT**: Behavioral therapy; **CABG**: coronary artery bypass grafting; **CaU**: Care as usual; **CBT**: Cognitive-behavioral therapy; **COPD:** Obstructive pulmonary disease; **CRQ**: Chronic Respiratory Questionnaire; **EADL**: Extended activities of daily living; **EQ**: Quality of Evidence; **ESD**: Early supported discharge service; **FACT**: Functional Assessment of Cancer Therapy; **FEV1**: Forced expiratory volume at 1s; **FU**: follow-up; **Ghb**: Glycated hemoglobin; **HAMD**: Hamilton Depression Rating Scale; **HbA1c**: Haemoglobin A1c; **HDL**: High-density lipoproteins; **HDRS:** Hamilton Depression Rating Scale; **HR**: Hazard Ratio; **hrs**: hours; **m**: months; **MD**: Mean difference; **min**: minutes; **MLWHF**: Minnesota Living With Heart Failure questionnaire; **MOS SF‐36**: Medical Outcomes 36‐Item Short Form Health Survey; **OR**: Odds Ratio; **PASfSP**: Postural Assessment Scale for Stroke Patients; **PCI**: Percutaneous coronary intervention; **PCP**: Physical conditioning program; **PEF**: Peak expiratory flow; **PHQ**: Patient health questionnaire; **PUFA**: Polyunsaturated fatty acids; **QLQ-C30**: European Organisation for Research and Treatment of Cancer Quality of Life Questionnaire-C30; **QoL**: Quality of Life; **RCT**: Randomized controlled trials; **RD**: Risk Difference; **RR**: Risk Ratio; **s**: seconds; **SF-36**: Short-Form-36 Health Survey; **SMD**: Standard mean difference; **TUG**: Timed Up and Go; **vs.**: versus; **w**: weeks; **y**: year

**E-Table 1: Hypertension**

| **Short Title** | **Independence and Functional Health** | **Symptom reduction, Mortality, Utilisation/Costs, Adverse Events, Others** |
| --- | --- | --- |
| 1. Semlitsch 2016: Long-term effects of weight-reducing diets in people with hypertension. CD008274 |  | Symptom reduction  Weight-reducing diet vs. No weight-reducing diet   - Systolic blood pressure: MD -4.49 [-7.20, -1.78]; EQ: low - Diastolic blood pressure: MD -3.19 [-4.83, -1.54]; EQ: low - Body weight: MD ‐3.98 [‐4.79, ‐3.17]; EQ: moderate |

**E-Table 2:** **Cardiovascular diseases incl. stroke**

| **Cardiovascular diseases** |  |  |
| --- | --- | --- |
| **Short Title** | **Independence and Functional Health** | **Symptom reduction, Mortality, Utilisation/Costs, Adverse Events, Others** |
| 1. Abdelhamid 2018: Polyunsaturated fatty acids for the primary and secondary prevention of cardiovascular disease. CD012345 |  | Symptom reduction  Higher Polyunsaturated fatty acids (PUFA) vs. lower PUFA - dichotomous secondary outcomes   - Myocardial infarction: RR 0.88 [0.78, 0.99]; EQ: -   Higher PUFA vs. lower PUFA - continuous secondary outcomes   - Serum total cholesterol: MD ‐0.12 [‐0.23, ‐0.02]; EQ: high - Serum fasting triglyceride: MD -0.12 [-0.20, -0.04]; EQ: moderate - Body weight: MD 0.76 [0.34. 1.19]; EQ: moderate |
| 1. Andersen 2017: Patient education in the management of coronary heart disease. CD008895 |  | Symptom reduction  Education vs. no education   - Other fatal and/or non-fatal cardiovascular events (FU: 21m): RR 0.36 [0.23, 0.56]; EQ: low |
| 1. Anderson 2017a: Home-based versus centre-based cardiac rehabilitation. CD007130 |  | Symptom reduction  Home-based vs. Centre based cardiac rehabilitation   - High-density lipoproteins (HDL) cholesterol (FU: 3-12m): MD -0.07 [-0.11, -0.03]; EQ: - - Triglycerides (FU: 3-12m): MD 0.15 [0.00, 0.29]; EQ: - |
| 1. Anderson 2017b: Exercise-based cardiac rehabilitation in heart transplant recipients. CD012264 |  | Symptom reduction  Exercise vs. No exercise   - Exercise capacity (VO_2_ peak): MD 2.49 [1.63, 3.36]; EQ: moderate |
| 1. Barth 2015: Psychosocial interventions for smoking cessation in patients with coronary heart disease. CD006886 |  | Others  Efficacy of psychosocial interventions on abstinence all trials vs. Usual care   - Abstinence (FU: 6-12m) (ITT preferred over completer): RR 1.24 [1.14, 1.35]; EQ: -   Efficacy of psychosocial interventions on abstinence without outliers vs. Usual care   - Abstinence (FU: 6-12m) (ITT preferred over completer): RR 1.22 [1.13, 1.32]; EQ: - |
| 1. Driscoll 2015: Nurse-led titration of angiotensin converting enzyme inhibitors, beta-adrenergic blocking agents, and angiotensin receptor blockers for people with heart failure with reduced ejection fraction. CD009889 |  | Mortality  Nurse-led titration vs. Usual care   - All-cause mortality (FU: 12m): RR 0.66 [0.48, 0.92]; EQ: moderate - All-cause event free survival (FU: 12m): RR 0.60 [0.46, 0.77]; EQ: moderate   Utilisation/Costs  Nurse-led titration vs. Usual care   - All-cause hospital admissions (FU: 12m): RR 0.80 [0.72, 0.88]; EQ: high - Heart failure-related hospital admissions (FU: 12m): RR 0.51 [0.36, 0.72]; EQ: moderate   Others  Nurse-led titration vs. Usual care   - Proportion reaching target dose of medications (FU: 12m): RR 1.99 [1.61, 2.47]; EQ: low |
| 1. Ebrahim 2011: Multiple risk factor interventions for primary prevention of coronary heart disease. CD001561 |  | Symptom reduction  Multiple risk factor intervention vs. Control   - Smoking prevalence: OR 0.87 [0.75, 1.00]; EQ: - - Systolic blood pressure: MD -3.38 [-3.63, -3.13]; EQ: - - Diastolic blood pressure: MD -2.41 [-2.55, -2.26]; EQ: - - Blood cholesterol: MD ‐0.07 [‐0.08, ‐0.06]; EQ: -   Mortality  Multiple risk factor intervention vs. Control   - Stroke mortality: OR 0.75 [0.60, 0.95]; EQ: - - Fatal or non-fatal clinical events: OR 0.84 [0.73, 0.98]; EQ: - |
| 1. Hulzebos 2012: Preoperative physical therapy for elective cardiac surgery patients. CD010118 |  | Symptom reduction  Preoperative physical therapy vs. No preoperative physical therapy   - Postoperative Pulmonary Complications grade 2 (atelectasis): RR 0.52 [0.32, 0.87]; EQ: - - Postoperative Pulmonary Complications grade 3 (pneumonia): RR 0.45 [0.24, 0.83]; EQ: -   Utilisation/Costs  Preoperative physical therapy vs. No preoperative physical therapy   - Length of postoperative hospital stay (days): MD ‐3.21 [‐5.73, ‐0.69]; EQ: - |
| 1. Inglis 2015: Structured telephone support or non‐invasive telemonitoring for patients with heart failure. CD007228 |  | Mortality  Structured telephone support vs. Usual care   - All-cause mortality: RR 0.87 [0.77, 0.98]; EQ: moderate   Telemonitoring vs. Usual care   - All-cause mortality: RR 0.80 [0.68, 0.94]; EQ: moderate   Utilisation/Costs  Structured telephone support vs. Usual care   - Chronic heart failure-related hospitalizations: RR 0.85 [0.77, 0.93]; EQ: moderate   Telemonitoring vs. Usual care   - Chronic heart failure-related hospitalizations: RR 0.71 [0.60, 0.83]; EQ: moderate |
| 1. Long 2018: Exercise-based cardiac rehabilitation for adults with stable angina. CD012786 |  | Symptom reduction  Exercise vs. No exercise for stable angina   - Exercise capacity (FU: 6-12m): SMD 0.45 [0.20, 0.70]; EQ: low   Utilisation/Costs  Exercise vs. No exercise for stable angina   - Revascularization procedure (Coronary artery bypass grafting (CABG) or percutaneous coronary intervention (PCI)): RR 0.27 [0.11, 0.64]; EQ: - |
| 1. Long 2019: Exercise-based cardiac rehabilitation for adults with heart failure. CD003331 | All exercise interventions vs. Usual care   - Health-related QoL - MLWHF (FU: 6-12m): MD -7.11 [-10.49, -3.73]; EQ: low - Health-related QoL - MLWHF and other scales (FU: 6-30m): SMD -0.60 [-0.82, -0.39]; EQ: low - Health‐related QoL ‐ MLWHF (FU: >12 m): MD ‐9.49 [‐17.48, ‐1.50]; EQ: - | Utilisation/Costs  All exercise interventions vs. Usual care   - Hospital admission (FU: 6-12m): RR 0.70 [0.60, 0.83]; EQ: moderate - Hospital admission heart failure only: RR 0.59 [0.42, 0.84]; EQ: low |
| 1. Nielsen 2019: Exercise-based cardiac rehabilitation for adult patients with an implantable cardioverter defibrillator. CD011828 |  | Symptom reduction  Exercise‐based cardiac rehabilitation vs. Control   - Exercise capacity (VO_2_ peak) (random-effects model) (FU: end of the intervention): MD 2.27 [1.07, 3.46]; EQ: - - Exercise capacity (VO_2_ peak) (fixed‐effect model) (FU: end of the intervention): MD 0.91 [0.60, 1.21]; EQ: -   Mortality  Exercise‐based cardiac rehabilitation vs. Control (best‐worst case meta‐analysis)   - All‐cause mortality (FU: end of the intervention): RR 0.07 [0.02, 0.22]; EQ: -   Exercise‐based cardiac rehabilitation vs. Control (worst‐best case meta‐analysis)   - All-cause mortality (fixed-effect model) (FU: end of the intervention): RR 16.78 [4.32, 65.18]; EQ: - - All-cause mortality (FU: end of the intervention): RR 13.50 [3.24, 56.25]; EQ: -   Exercise‐based cardiac rehabilitation vs. Control (best‐worst case meta‐analysis)   - All‐cause mortality (at longest FU): RR 0.48 [0.32, 0.74]; EQ: -   Exercise‐based cardiac rehabilitation vs. Control (worst‐best case meta‐analysis)   - All‐cause mortality (at longest FU): RR 2.29 [1.12, 4.70]; EQ: -   Adverse Events  Exercise‐based cardiac rehabilitation vs. Control   - Non‐serious adverse events (FU: end of the intervention): RR 5.44 [1.01, 29.32]; EQ: -   Exercise‐based cardiac rehabilitation vs. Control (best‐worst case meta‐analysis)   - Serious adverse events (FU: end of the intervention): RR 0.58 [0.41, 0.82]; EQ: -   Exercise‐based cardiac rehabilitation vs. Control (worst‐best case meta‐analysis)   - Serious adverse events (FU: end of the intervention): RR 1.74 [1.34, 2.27]; EQ: -   Exercise‐based cardiac rehabilitation vs. Control (best‐worst case meta‐analysis)   - Serious adverse events (at longest FU): RR 0.48 [0.32, 0.74]; EQ: -   Exercise‐based cardiac rehabilitation vs. Control (worst‐best case meta‐analysis)   - Serious adverse events (fixed‐effect model) (at longest FU): RR 0.71 [0.55, 0.93]; EQ: - |
| 1. Richards 2017: Psychological interventions for coronary heart disease. CD002902 | Psychological intervention (alone or with other rehabilitation) vs. Comparator (usual care or other rehabilitation)   - Depression (FU: 12m): SMD -0.27 [-0.39, -0.15]; EQ: low - Anxiety (FU: 12m): SMD ‐0.24 [‐0.38, ‐0.09]; EQ: low | Symptom reduction  Psychological intervention (alone or with other rehabilitation) vs. Comparator (usual care or other rehabilitation)   - Stress (FU: 12m): SMD ‐0.56 [‐0.88, ‐0.24]; EQ: very low   Mortality  Psychological intervention (alone or with other rehabilitation) vs. Comparator (usual care or other rehabilitation)   - Cardiac mortality (FU: 57m): RR 0.79 [0.63, 0.98]; EQ: low |
| 1. Risom 2017: Exercise-based cardiac rehabilitation for adults with atrial fibrillation. CD011197 | Exercise vs. No exercise   - Quality of Life (QoL), Short-Form-36 Health Survey (SF-36), General Health (FU: 20w-6m): MD 7.11 [3.46, 10.77]; EQ: low - QoL, SF-36, Vitality (FU: 20w-6m): MD 6.10 [1.91, 10.30]; EQ: low - Exercise capacity (6mWT) (FU: 12w-4mo): MD 75.76 [14.00, 137.53]; EQ: very low | Symptom reduction  Exercise vs. No exercise   - Exercise capacity (VO_2_ peak) (FU: 4-5m): MD 3.76 [1.37, 6.15]; EQ: moderate - Exercise capacity (SMD): SMD 0.86 [0.46, 1.26]; EQ: very low |
| 1. Santiago de Araújo Pio 2019: Interventions to promote patient utilisation of cardiac rehabilitation. CD007131 |  | Utilisation of Cardiac Rehabilitation (UCR)  Promoting UCR vs. not promoting   - Enrolment, RR 1.27 [1.13, 1.42], EQ: low - Adherence, SMD 0.38 [0.20, 0.55], EQ: low - Completion, RR 1.13 [1.02, 1.25], EQ: moderate |
| 1. Sibilitz 2016: Exercise-based cardiac rehabilitation for adults after heart valve surgery. CD010876 |  | Symptom reduction  Exercise vs. No exercise   - Exercise capacity (bicycle exercise, metabolic equivalents/kilo joules) (FU: 3-6m, end of intervention): SMD ‐0.47 [‐0.81, ‐0.13]; EQ: moderate - Exercise capacity (bicycle exercise, metabolic equivalents/kilo joules) (at longest FU): SMD ‐0.50 [‐0.85, ‐0.14]; EQ: - |
| 1. Takeda 2019: Disease management interventions for heart failure. CD002752* | Multidisciplinary vs. Usual care   - QoL ‐ MLHFQ: MD ‐12.21 [‐16.43, ‐7.99]; EQ: very low | Utilisation/Costs  Case management vs. Usual care   - Heart failure readmissions ‐ main analysis: RR 0.64 [0.53, 0.78]; EQ: moderate   Multidisciplinary vs. Usual care   - Heart failure readmissions ‐ main analysis: RR 0.68 [0.50, 0.92]; EQ: -   Mortality  Case management vs. Usual care   - All‐cause mortality ‐ main analysis: RR 0.78 [0.68, 0.90]; EQ: low   Multidisciplinary vs. Usual care   - Heart failure mortality - main analysis: RR 0.46 [0.23, 0.95]; EQ: - - All‐cause mortality ‐ main analysis: RR 0.67 [0.54, 0.83]; EQ: - |
| 1. Taylor 2019: Exercise‐based rehabilitation for adults with heart failure. CD003331 | All exercise interventions vs. Usual care   - Health‐related QoL - Minnesota Living With Heart Failure questionnaire (MLWHF) (FU: 6-12m): MD ‐7.11 [‐10.49, ‐3.73]; EQ: low - Health‐related QoL - MLWHF and other scales (FU: 6-30m): SMD ‐0.60 [‐0.82, ‐0.39]; EQ: low - Health‐related QoL - MLWHF (FU: >12m): MD ‐9.49 [‐17.48, ‐1.50]; EQ: - | Utilisation/Costs  All exercise interventions vs. Usual care:   - Hospital admission (FU: ≤ 12m): RR 0.70 [0.60, 0.83]; EQ: moderate - Hospital admission heart failure only: RR 0.59 [0.42, 0.84]; EQ: low |
| **Stroke** |  |  |
| **Short Title** | **Independence and Functional Health** | **Symptom reduction, Mortality, Utilization/Costs, Adverse Events, Others** |
| 1. Bridgwood 2018: Interventions for improving modifiably risk factor control in the secondary prevention of stroke. CD009103 |  | Symptom reduction  Organisational interventions vs. Usual care   - Blood pressure target achievement: OR 0.70 [0.53, 0.92]; EQ: moderate - Mean low density lipoprotein: MD ‐0.19 [‐0.30, ‐0.09]; EQ: moderate   Mortality  Organisational interventions vs. Usual care   - Number of vascular deaths: OR 0.38 [0.15, 0.97]; EQ: - |
| 1. Coupar 2010: Simultaneous bilateral training for improving arm function after stroke. CD006432 | Bilateral training vs. Usual care   - Subtotal: Motor impairment of the upper limb, Motor impairment scales: SMD 0.43 [0.06, 0.81]; EQ: - |  |
| 1. English 2017: Circuit class therapy for improving mobility after stroke. CD007513 | Circuit class therapy vs. Other   - 6-min Walking Test (6mWT): MD 60.86 [44.55, 77.17]; EQ: moderate - Gait speed: MD 0.15 [0.10, 0.19]; EQ: moderate - Cadence: MD 13.57 [7.52, 19.62]; EQ: - - Timed Up and Go (TUG): MD -3.62 [-6.09, -1.16]; EQ: low - Rivermead Mobility Index: MD 0.56 [0.17, 0.95]; EQ: - - Functional Ambulation Classification: OR 1.91 [1.01, 3.60]; EQ: moderate - Activities-specific Balance Scale: MD 7.76 [0.66, 14.87]; EQ: - - Stroke Impact Scale (physical): MD 2.91 [0.00, 5.82]; EQ: low - Steps per Day: MD 1325.66 [411.09, 2240.22]; EQ: - | Symptom reduction  Circuit class therapy vs. Other   - VO_2_ peak: MD 2.81 [0.90, 4.72]; EQ: -   Others  Circuit class therapy + Education vs. No therapy   - Carer Strain Index: MD 1.06 [0.39, 1.73]; EQ: - |
| 1. French 2016: Repetitive task training for improving functional ability after stroke. CD006073 | Repetitive task training vs. Usual care, attention control or no treatment  Upper limb function   - Arm function (FU: post treatment): SMD 0.25 [0.01, 0.49]; EQ: low - Hand function (FU: post treatment): SMD 0.25 [0.00, 0.51]; EQ: low - Sitting balance/reach (FU: post treatment): SMD 0.28 [0.01, 0.55]; EQ: - - Subtotal: Upper limb function, FU: post treatment <6m: SMD 0.92 [0.58, 1.26]; EQ: -   Lower limb function   - Walking distance (FU: post treatment): MD 34.80 [18.19, 51.41]; EQ: moderate - Functional ambulation (FU: post treatment): SMD 0.35 [0.04, 0.66] ; EQ: moderate - Sit-to-stand (FU: post treatment): SMD 0.35 [0.13, 0.56]; EQ: - - Lower limb functional measures (FU: post treatment): SMD 0.29 [0.10, 0.48]; EQ: low - Standing balance/reach (FU: post treatment): SMD 0.24 [0.07, 0.42]; EQ: - - Subtotal: Lower limb function, FU: post treatment <6m: SMD 0.34 [0.16, 0.52]; EQ: -   Secondary outcomes   - ADL function: SMD 0.28 [0.10, 0.45]; EQ: - - Global motor function scales: SMD 0.38 [0.11, 0.65]; EQ: moderate - QoL/health status: SMD 0.28 [0.04, 0.53]; EQ: - | Adverse Events   - narratively reported |
| 1. Fryer 2016: Self-management programmes for quality of life in people with stroke. CD010442 | Self-management vs. Control   - QoL: SMD 0.20 [-0.00, 0.41]; EQ: low | Symptom reduction  Self-management vs. Control   - Subtotal: Self-efficacy, Stroke Self‐Efficacy Questionnaire: SMD 0.33 [0.04, 0.61]; EQ: low |
| 1. Langhorne 2017: Early supported discharge services for people with acute stroke. CD000443 | Early supported discharge service (ESD) vs. Conventional care: patient outcomes   - Extended activities of daily living (EADL) score: SMD 0.14 [0.03, 0.25]; EQ: low | Mortality  ESD vs. Conventional care: patient outcomes   - Subtotal: Death, No ESD team: OR 2.14 [1.19, 3.85]; EQ: - - Death or dependency: OR 0.80 [0.67, 0.95]; EQ: moderate - Death or dependency (within 6m): OR 0.70 [0.56, 0.87]; EQ: -   Utilisation/Costs  ESD vs. Conventional care: patient outcomes   - Death or requiring institutional care: OR 0.75 [0.59, 0.96]; EQ: moderate - Length of initial hospital stay: MD ‐5.54 [‐8.18, ‐2.91]; EQ: moderate   Others  ESD vs. Conventional care: patient outcomes   - Satisfaction with services: OR 1.60 [1.08, 2.38]; EQ: low |
| 1. Mant 2010: Stroke liaison workers for stroke patients and carers: an individual patient data meta‐analysis. CD005066 |  | Others  Stroke liaison workers vs. Usual care: intervention type   - 'I have been treated with kindness and respect': patient: OR 0.53 [0.28, 1.00]; EQ: - - 'Someone has really listened': patient: OR 1.58 [1.14, 2.19]; EQ: - - 'I received all the information I needed about the nature and causes of the patient's illness': carer: OR 1.72 [1.04, 2.85]; EQ: - - 'I have received enough information about recovery and rehabilitation': carer: OR 1.98 [1.25, 3.14]; EQ: - - 'Someone has really listened': carer, OR 2.56 [1.52, 4.31]; EQ: - - 'I have not felt neglected': carer: OR 2.62 [1.44, 4.77]; EQ: - |
| 1. Mehrholz 2017a: Treadmill training and body weight support for walking after stroke. CD002840 | Treadmill (with or without body weight support) vs. Other intervention   - Walking speed (minutes (min)/seconds (s)) (FU: end of treatment): MD 0.06 [0.03, 0.09]; EQ: moderate - Walking endurance (min) (FU: end of treatment): MD 14.19 [2.92, 25.46]; EQ: moderate   Treadmill and body weight support vs. Other interventions   - Walking speed (min/s) (FU: end of treatment): MD 0.07 [0.02, 0.11]; EQ: - - Walking endurance (min) (FU: end of treatment): MD 20.79 [0.43, 41.14]; EQ: -   Treadmill training without body weight support vs. Other interventions   - Subtotal: Walking speed (min/s) (FU: end of treatment), Independent in walking at start of treatment (min/s): MD 0.05 [0.01, 0.09]; EQ: - |  |
| 1. Mehrholz 2017b: Electromechanical-assisted training for walking after stroke. CD006185 | Electromechanical- and robotic-assisted gait training plus physiotherapy vs. Physiotherapy (or usual care)   - Independent walking, all electromechanical devices used (FU: end of intervention phase): OR 1.94 [1.39, 2.71]; EQ: moderate |  |
| 1. Mehrholz 2018: Electromechanical and robot-assisted arm training after stroke. CD006876 | Electromechanical and robotic assisted training vs. Other intervention   - ADL (FU: end of intervention phase): SMD 0.31 [0.09, 0.52]; EQ: high - ADL - participants treated in the acute and subacute phase of their stroke (FU: end of intervention phase): SMD 0.40 [0.10, 0.70]; EQ: high - Arm function (FU: end of intervention phase): SMD 0.32 [0.18, 0.46]; EQ: high - Arm muscle strength (FU: end of intervention phase): SMD 0.46 [0.16, 0.77]; EQ: high |  |
| 1. Pollock 2014a: Interventions for improving sit-to-stand ability following stroke. CD007232 | Specific sit-to-stand training vs. Control   - Time (immediate): SMD -0.34 [-0.62, -0.06]; EQ: moderate - Lateral symmetry (immediate): SMD 0.85 [0.38, 1.33], moderate - Time (FU): SMD -0.45 [-0.78, -0.12]; EQ: - - Lateral symmetry (FU): SMD 0.59 [0.11, 1.07]; EQ: - |  |
| 1. Pollock 2014b: Physical rehabilitation for the recovery of function and mobility following stroke. CD001920 | Intervention vs. No treatment   - Independence in ADL scales (immediate): SMD 0.78 [0.58, 0.97]; EQ: moderate - Motor function scales (immediate): SMD 0.81 [0.58, 1.04]; EQ: moderate - Independence in ADL scales (persisting): SMD 0.58 [0.11, 1.04]; EQ: moderate - Motor function scales (persisting): SMD 1.06 [0.37, 1.75]; EQ: moderate   Intervention vs. Usual care or attention control   - Motor function scales (immediate): SMD 0.42 [0.24, 0.61]; EQ: moderate - Balance (BBS) (immediate): SMD 0.31 [0.05, 0.56]; EQ: moderate - Gait velocity (immediate): SMD 0.46 [0.32, 0.60]; EQ: high - Gait velocity (persistent): SMD 0.38 [0.10, 0.66]; EQ: moderate |  |
| 1. States 2009: Overground physical therapy gait training for chronic stroke patients with mobility deficits. CD006075 | Gait training vs. Control   - Gait speed (min/s) (FU: end of treatment): MD 0.07 [0.05, 0.10]; EQ: - - TUG (s) (FU: end of treatment): MD -1.81 [-2.29, -1.33]; EQ: - - 6mWT (min) (FU: end of treatment): MD 26.06 [7.14, 44.97]; EQ: - |  |
| 1. Thieme 2018: Mirror therapy for improving motor function after stroke. CD008449 | Mirror therapy vs. All other interventions   - Motor function All outcome measures (FU: end of intervention phase),: SMD 0.47 [0.27, 0.67]; EQ: moderate - Motor impairment All outcome measures (FU: end of intervention phase),: SMD 0.49 [0.32, 0.66]; EQ: moderate - Subtotal: Activities of daily living (ADL) (FU: end of intervention phase), All outcome measures: SMD 0.48 [0.30, 0.65]; EQ: moderate - Subtotal: Pain (FU: end of intervention phase), All outcome measures: SMD -0.89 [-1.67, -0.11]; EQ: low - Subtotal: Motor impairment (FU: 6m), All outcome measures: SMD 0.69 [0.26, 1.12]; EQ: - |  |
| 1. Vloothuis 2016: Caregiver-mediated exercised for improving outcomes after stroke. CD011058 | Caregiver-mediated exercised vs. Control   - Gait and gait-related measures: balance: combined (Berg Balance Scale (BBS) (FU: end of intervention), Postural Assessment Scale for Stroke Patients (PASfSP)): SMD 0.53 [0.19, 0.87]; EQ: - - Measures of upper limb activities function: Wolf Motor Function Test - performance time (FU: end of intervention): MD ‐1.72 [‐2.23, ‐1.21]; EQ: - - Subtotal: Patient: ADL measures (combined) for patients with timing post stroke <6m, FU: end of intervention: SMD 0.44 [0.01, 0.86]; EQ: - |  |

**E-Table 3: Polypharmacy**

| **Short Title** | **Independence and Functional Health** | **Symptom reduction, Mortality, Utilisation/Costs, Adverse Events, Others** |
| --- | --- | --- |
| 1. Rankin 2018: Interventions to improve the appropriate use of polypharmacy for older people. CD008165 * |  | Others  Pharmaceutical care vs. Usual care (postintervention analysis)   - Medication appropriateness (as measured by an implicit tool): MD -4.76 [-9.20, -0.33]; EQ: very low - The number of potentially inappropriate medications: SMD -0.22 [-0.38, -0.05]; EQ: very low - The number of potential prescribing omissions: SMD -0.81 [-0.98, -0.64]; EQ: low - The proportion of patients with one or more potential prescribing omissions: RR 0.40 [0.18, 0.85]; EQ: very low |
| 1. van Driel 2016: Interventions to improve adherence to lipid-lowering medication. CD004371 |  | Symptom reduction  Intensified patient care vs. Usual care   - Reduction in total serum cholesterol (mg/dL) (FU: ≤6m): MD 17.15 [1.17, 33.14]; EQ: low - Reduction in total serum cholesterol (mg/dL) (FU: >6m): MD 17.57 [14.95, 20.19]; EQ: high - Reduction in LDL‐C (mg/dL) (FU: ≤6m): MD 19.51 [8.51, 30.51]; EQ: moderate   Others  Intensified patient care vs. Usual care   - Medication adherence (FU: ≤6m): OR 1.93 [1.29, 2.88]; EQ: moderate - Medication adherence (FU: >6m): OR 2.87 [1.91, 4.29]; EQ: high |

**E-Table 4: Osteoarthritis**

| **Short Title** | **Independence and Functional Health** | **Symptom reduction, Mortality, Utilisation/Costs, Adverse Events, Others** |
| --- | --- | --- |
| 1. Hurley 2018: Exercise interventions for people with hip or knee osteoarthritis. CD010842 | Exercise vs. Control   - Pain: SMD -0.20 [-0.28, -0.11]; EQ: - - Subtotal: Pain, Western Ontario and McMaster Universities Osteoarthritis Index Pain (FU: 12w-30m): SMD -0.33 [-0.46, -0.21]; EQ: moderate - Physical function (FU: 9w-30m): SMD -0.27 [-0.37, -0.17]; EQ: moderate - Depression (FU: 8w-30m): SMD -0.16 [-0.29, -0.02]; EQ: moderate - SF-36 mental health: MD 5.07 [2.43, 7.72]; EQ: - - SF-36 vitality: MD 6.06 [3.57, 8.54]; EQ: - | Symptom reduction  Exercise vs. Control   - Self-efficacy (FU: 12w-18m): SMD 0.46 [0.34, 0.58]; EQ: low - Stress: MD ‐4.76 [‐7.57, ‐1.95]; EQ: - |
| 1. Østerås 2017: Exercise for hand osteoarthritis. CD010388 | Exercise vs. No exercise (FU: short-term)   - Hand pain (FU: 3m): SMD -0.27 [-0.47, -0.07]; EQ: low - Finger joint stiffness (FU: 3m): SMD -0.36 [-0.58, -0.15]; EQ: low - Osteoarthritis Research Society International/Outcome Measures/Outcome Measures in Rheumatology responder criteria (Pain): RR 2.80 [1.40, 5.62]; EQ: - |  |

**E-Table 5: Back Pain**

| **Short Title** | **Independence and Functional Health** | **Symptom reduction, Mortality, Utilisation/Costs, Adverse Events, Others** |
| --- | --- | --- |
| 1. French 2006: Superficial heat or cold for low back pain. CD004750 | Heat versus (vs.) placebo or non‐heated wrap (acute and sub‐acute low back pain < 3 months (m))   - Subtotal: Pain relief (higher score favours heat), Follow-up (FU): ≤ day 5: Mean Difference (MD) 1.06 [0.68, 1.45]; Quality of Evidence (EQ): - - Subtotal: Function (lower score favours heat), FU: day 4: MD ‐2.12 [‐3.07, ‐1.18]; EQ: - |  |
| 1. Kamper 2014: Multidisciplinary biopsychosocial rehabilitation for chronic low back pain. CD000963 | Multidisciplinary biopsychosocial rehabilitation (MBR) vs. usual care   - Back pain (FU: Short-term): Standard Mean Difference (SMD) -0.55 [-0.83, -0.28]; EQ: - - Back pain (FU: Medium-term): SMD -0.60 [-0.85, -0.34]; EQ: - - Back pain (FU: Long-term): SMD -0.21 [-0.37, -0.04]; EQ: moderate - Disability (FU: Short-term): SMD -0.41 [-0.62, -0.19]; EQ: - - Disability (FU: Medium-term): SMD -0.43 [-0.66, -0.19]; EQ: - - Disability (FU: Long-term): SMD -0.23 [-0.40, -0.06]; EQ: moderate - QoL SF36 Mental Component Summary (MCS) (FU: Short-term): MD 15.25 [2.05, 28.44]; EQ - - QoL SF36 Mental Component Summary (FU: Medium-term): MD 7.59 [1.69, 13.49]; EQ: -   MBR vs. physical treatment   - Pain (FU: Short-term): SMD -0.30 [-0.54, -0.06]; EQ: - - Pain (FU: Medium-term): SMD -0.28 [-0.54, -0.02]; EQ: - - Disability (FU: Short-term): SMD -0.39 [-0.68, -0.10]; EQ: - - Disability (FU: Long-term): SMD -0.68 [-1.19, -0.16]; EQ: low   MBR vs. wait list   - Pain (FU: Short-term): SMD -0.73 [-1.22, -0.24]; EQ: - - Disability (FU: Short-term): SMD ‐0.49 [‐0.76, ‐0.22]; EQ: - | Symptom reduction  MBR vs. usual care   - Catastrophising (FU: Short-term): SMD -0.43 [-0.83, -0.03]; EQ: - - Catastrophising (FU: Long-term): SMD -0.40 [-0.76, -0.05]; EQ: - Fear avoidance (FU: Long-term): SMD -0.29 [-0.49, -0.08]; EQ: -   Utilisation/Costs  MBR vs. physical treatment   - Work (FU: Medium-term): OR 2.14 [1.12, 4.10]; EQ: - - Work (FU: Long-term): OR 1.87 [1.39, 2.53]; EQ: moderate   Adverse Events   - Adverse events/complications: OR 28.25 [3.77, 211.93]; EQ: low   Others  MBR vs. physical treatment   - Coping (FU: Medium-term): SMD 1.09 [0.31, 1.87]; EQ: - - Coping (FU: Long-term): SMD 0.30 [0.06, 0.54]; EQ: - |
| 1. Marin 2017: Multidisciplinary biopsychosocial rehabilitation for subacute low back pain. CD002193 | Multidisciplinary rehabilitation vs. usual care   - Subtotal: Pain, FU: Short-term: SMD -0.40 [-0.74, -0.06]; EQ: - - Subtotal: Pain, FU: Long-term: SMD -0.46 [-0.70, -0.21]; EQ: moderate - Subtotal: Disability, FU: Short-term: SMD -0.38 [-0.63, -0.14]; EQ: - - Subtotal: Disability, FU: Long-term: SMD ‐0.44 [‐0.87, ‐0.01]; EQ: low | Utilisation/Costs  Multidisciplinary rehabilitation vs. Usual care   - Return to work: OR 3.19 [1.46, 6.98]; EQ: very low - Sick leave periods: SMD ‐0.38 [‐0.66, ‐0.10]; EQ: low |
| 1. Saragiotto 2016: Motor control exercise for chronic non-specific low back pain. CD012004 | Motor control exercise vs. other exercises   - Subtotal: Pain, FU: Short-term < 3m: MD -7.53 [-10.54, -4.52]; EQ: low - Subtotal: Disability, FU: Short-term < 3m: MD -4.82 [-6.95, -2.68]; EQ: low - Subtotal: Function, FU: Short-term < 3m: MD 7.29 [1.53, 13.04]; EQ: -   Motor control exercise vs. Minimal intervention   - Subtotal: Pain, FU: Short-term < 3m: MD -10.01 [-15.67, -4.35]; EQ: moderate - Subtotal: Pain, FU: Intermediate ≥ 3-12m: MD ‐12.61 [‐20.53, ‐4.69]; EQ: low - Subtotal: Pain, FU: Long-term >12m: MD -12.97 [-18.51, -7.42]; EQ: moderate - Subtotal: Disability, FU: Short-term < 3m: MD -8.63 [-14.78, -2.47]; EQ: very low - Subtotal: Disability, FU: Intermediate ≥ 3-12m: MD -5.47 [-9.17, -1.77]; EQ: moderate - Subtotal: Disability, FU: Long-term >12m: MD -5.96 [-9.81, -2.11]; EQ: moderate   Motor control exercise vs. Exercise and electrophysical agents   - Subtotal: Pain, Short-term < 3m: MD ‐30.18 [‐35.32, ‐25.05]; EQ: low - Subtotal: Pain, Intermediate ≥ 3-12m: MD ‐19.39 [‐36.83, ‐1.96]; EQ: very low |  |
| 1. Schaafsma 2013: Physical conditioning as part of a return to work strategy to reduce sickness absence for workers with back pain. CD001822 |  | Utilisation/Costs  Intense physical conditioning program (PCP) + Care as usual (CaU) vs. CaU only, subacute pain   - Time to return to work (FU: Very long-term), SMD ‐0.39 [‐0.76, ‐0.02]; EQ: -   Intense PCP vs. Multidisciplinary exercise treatment, subacute pain   - Proportion off work (FU: Short-term 3-12m): OR 0.58 [0.42, 0.80]; EQ: - - Subtotal: Proportion off work, FU: 12m: OR 0.63 [0.40, 0.99]; EQ: -   Intense PCP vs. CaU, chronic pain   - Subtotal: Time to return to work, FU: 12m: SMD ‐0.23 [‐0.42, ‐0.03]; EQ: - |
| 1. Wieland 2017: Yoga treatment for chronic non-specific low back pain. CD010671 | Yoga vs. Non‐exercise control   - Subtotal: Back-specific function, FU: 4-6 weeks (w): SMD -0.45 [-0.71, -0.19]; EQ: low - Subtotal: Back-specific function, FU: 3-4m: SMD -0.40 [-0.66, -0.14]; EQ: low - Subtotal: Back-specific function, FU: 6m: SMD -0.44 [-0.66, -0.22]; EQ: moderate - Subtotal: Back-specific function, FU: 12m: SMD -0.26 [-0.46, -0.05]; EQ: low - Subtotal: Pain, FU: 4-6w: MD -10.83 [-20.85, -0.81]; EQ: very low - Subtotal: Pain, FU: 3-4m: MD -4.55 [-7.04, -2.06]; EQ: moderate - Subtotal: Pain, FU: 6m: MD -7.81 [-13.37, -2.25]; EQ: low - Subtotal: Clinical improvement, FU: 4-6w: Risk Ratio (RR) 2.62 [1.22, 5.67]; EQ: - - Subtotal: Clinical improvement, FU: 3m: RR 3.18 [1.86, 5.44]; EQ: - |  |

**E-Table 6: Incontinence**

|  |  |  |
| --- | --- | --- |
| 1. Clement 2013: Urodynamic studies for management of urinary incontinence in children and adults. CD003195 |  | Symptom reduction  Urodynamics vs. Clinical management   - Subtotal: Health status measures, Change in urinary incontinence (Urogenital Distress Inventory) FU: 12m: MD 14.70 [7.21, 22.19]; EQ: -   Utilisation/Costs  Urodynamics vs. Clinical management   - Number whose treatment was changed after urodynamics: RR 5.07 [1.87, 13.74]; EQ: low   Others  Urodynamics vs. Clinical management   - Number treated with drugs: RR 2.09 [1.32, 3.31]; EQ: - |
| 1. Imamura 2015: Lifestyle interventions for the treatment of urinary incontinence in adults. CD003505 |  | Symptom reduction  Weight loss vs. No active intervention   - Subtotal: Improvement rates based on quantification of symptoms, FU: 12 m: RR 1.21 [1.02, 1.44]; EQ: low |
| 1. Lipp 2014: Mechanical devices for urinary incontinence in women. CD001756 |  | Symptom reduction  Intravaginal mechanical device vs. No treatment   - Subtotal: Formal pad weighing tests after exercise (grams), Pessary vs. no treatment: MD ‐6.55 [‐11.20, ‐1.90]; EQ: - |
| 1. Norton 2012: Biofeedback and/or sphincter exercises for the treatment of faecal incontinence in adults. CD002111 |  | Symptom reduction  Anal sphincter exercises/ Pelvic floor muscle training with or without biofeedback + another treatment vs. Exercises with or without biofeedback   - Subtotal: Number of people failing to achieve full continence (worse, unchanged or improved), Exercises + biofeedback + electrical stimulation vs. exercises + biofeedback alone: RR 0.60 [0.46, 0.78]; EQ: - |
| 1. Thomas 2019: Interventions for treating urinary incontinence after stroke in adults. CD004462 | Intervention vs. No intervention/usual care   - Subtotal: Functional ability - mean Barthel score (continuous variables), Physical therapy interventions: MD 8.97 [1.27, 16.68]; EQ: moderate | Symptom reduction  Intervention vs. No intervention/usual care   - Subtotal: Number of participants continent after treatment, Complementary therapy intervention: RR 2.82 [1.57, 5.07]; EQ: low - Subtotal: Number of incontinent episodes in 24 hours, Physical therapy interventions: MD ‐4.76 [‐8.10, ‐1.41]; EQ: low |

**E-Table 7: Diabetes Mellitus**

| **Short Title** | **Independence and Functional Health** | **Symptom reduction, Mortality, Utilization/Costs, Adverse Events, Others** |
| --- | --- | --- |
| 1. Attridge 2014: Culturally appropriate health education for people in ethnic minority groups with type 2 diabetes mellitus. CD006424 |  | Symptom reduction  Culturally tailored health education compared vs. Conventional or usual diabetes health care   - HbA1c (FU: 3-4m): MD ‐0.39 [‐0.64, ‐0.13]; EQ: - - HbA1c (FU: ≤ 6m): MD ‐0.53 [‐0.72, ‐0.35]; EQ: - - HbA1c (FU: ≤ 1y): MD ‐0.19 [‐0.34, ‐0.04]; EQ: - - HbA1c (FU: 24m): MD ‐0.33 [‐0.61, ‐0.06]; EQ: - - HbA1c (at all points): MD ‐0.30 [‐0.38, ‐0.22]; EQ: - - Total cholesterol (at all endpoints): MD ‐6.14 [‐11.45, ‐0.82]; EQ: - - Triglycerides (FU: 3-4m): MD ‐23.98 [‐39.73, ‐8.23]; EQ: - - Systolic blood pressure (at all endpoints): MD 1.68 [0.35, 3.02]; EQ: - - Diastolic blood pressure (FU: ≤ 6 m): 1.95 [0.62, 3.28]; EQ: -   Others  Culturally tailored health education compared vs. Conventional or usual diabetes health care   - Final mean knowledge (FU: ≤ 6m): SMD 0.50 [0.33, 0.68]; EQ: - - Final mean knowledge (FU: ≤ 3m): SMD 0.35 [0.10, 0.59]; EQ: - - Final mean knowledge (FU: 1y): SMD 0.35 [0.13, 0.57]; EQ: - - Final mean knowledge (at all points): MD 0.89 [0.39, 1.39]; EQ: - |
| 1. Baumeister 2012: Psychological and pharmacological interventions for depression in patients with diabetes mellitus and depression. CD00838 | Psychological intervention vs. Usual care/waiting list control   - Depression score (FU: Medium‐term): SMD ‐0.42 [‐0.70, ‐0.14]; EQ: - - Depression remission (FU: Short‐term): OR 2.88 [1.58, 5.25]; EQ: moderate - Depression remission (FU: Medium‐term): OR 2.49 [1.44, 4.32]; EQ: low   Pharmacological intervention vs. Placebo   - Depression score (FU: Short‐term): SMD ‐0.61 [‐0.94, ‐0.27]; EQ: - - Depression remission (FU: Short‐term): OR 2.50 [1.21, 5.15]; EQ: low | Symptom reduction  Pharmacological intervention vs. Placebo   - Glycaemic control (HbA1c) (FU: Short‐term): MD ‐0.36 [‐0.59, ‐0.13]; EQ: - |
| 1. Chew 2017: Psychological interventions for diabetes‐related distress in adults with type 2 diabetes mellitus. CD011469 | Cognition‐focused vs. usual care   - Self‐efficacy, brief and simple interventions, SMD 0.21 [0.04, 0.38] ]; EQ: - - Self‐efficacy, age ≥ 60 years, SMD 0.21 [0.04, 0.38] ]; EQ: - | Symptom reduction  Psychological interventions versus usual and enhanced diabetes care   - Diabetes‐related distress, SMD ‐0.07 [‐0.13, ‐0.00]; EQ: - - Diabetes‐related distress, SMD ‐0.07 [‐0.13, ‐0.00]; EQ: - - Self-efficacy, SMD 0.13 [0.00, 0.27]; EQ: -   Psychological interventions versus usual diabetes care   - Self-efficacy, SMD 0.20 [0.04, 0.37]; EQ: - - Self-efficacy, SMD 0.20 [0.04, 0.37]; EQ: - - HbA1c, MD ‐0.17 [‐0.33, ‐0.00]; EQ: -   Psychological interventions versus usual care (trials with low overall risk of bias)   - Self‐efficacy, SMD 0.30 [0.09, 0.51]; EQ: - |
| 1. Nield 2007: Dietary advice for treatment of type 2 diabetes mellitus in adults. CD004097 |  | Symptom reduction  Dietary Advice vs. Dietary Advice + Exercise   - Weight (FU: 12m): MD ‐6.74 [‐11.72, ‐1.76]; EQ: - - HbA1c (FU: 6m): MD ‐0.86 [‐1.33, ‐0.38]; EQ: - - HbA1c (FU: 12m): MD ‐0.96 [‐1.53, ‐0.39]; EQ: - |
| 1. Norris 2006: Long-term non-pharmacological weight loss interventions for adults with type 2 diabetes mellitus. CD004095 |  | Symptom reduction  Very-Low-Calorie Diet vs. Different intervention   - Fasting blood sugar: MD ‐1.42 [‐2.30, ‐0.54]; EQ:- (fixed) - Glycated hemoglobin (GHb): MD ‐1.03 [‐1.71, ‐0.36]; EQ: - (fixed) - Total Cholesterol: MD 0.26 [0.01, 0.51]; EQ: - (fixed) - HDL: MD 0.06 [0.00, 0.11]; EQ: - (fixed) - HDL: MD 0.06 [0.00, 0.11]; EQ:- (random)   Any intervention vs. Usual care   - Weight loss (kg): MD ‐1.91 [‐3.00, ‐0.82]; EQ: - (fixed) - GHb: MD ‐0.74 [‐0.99, ‐0.48]; EQ: - (fixed) - HDL: MD 0.11 [0.06, 0.17]; EQ: - (fixed) - Triglycerides: MD ‐0.36 [‐0.58, ‐0.14]; EQ: - (fixed) - Weight loss (%): MD ‐3.10 [‐4.48, ‐1.72]; EQ: - (fixed) - Weight loss (kg): MD ‐1.72 [‐3.15, ‐0.29]; EQ: - (random) - Triglycerides: MD ‐0.36 [‐0.58, ‐0.14]; EQ: - (random) - Weight loss (%): MD ‐3.10 [‐4.48, ‐1.72]; EQ: - (random)   Any intervention vs. Usual care (HbA1c in original paper)   - GHb (FU: 1‐2y): MD ‐0.76 [‐1.03, ‐0.50]; EQ: - (fixed model) |
| 1. Pal 2013: Computer-based diabetes self-management interventions for adults with type 2 diabetes mellitus. CD008776 |  | Symptom reduction  Computer-based interventions vs. Standard diabetes care   - HbA1c: MD ‐0.21 [‐0.37, ‐0.05]; EQ: -   Dietary change vs. Standard diabetes care   - Pooled effect on diet: SMD ‐0.29 [‐0.43, ‐0.15]; EQ: - |

**E-Table 8: Depression**

| **Short Title** | **Independence and Functional Health** | **Symptom reduction, Mortality, Utilization/Costs, Adverse Events, Others** |
| --- | --- | --- |
| 1. Archer 2012: Collaborative care for depression and anxiety problems. CD006525 | Collaborative care vs. Usual care   - Subtotal :Depression response, FU: 0-6m: RR 1.32 [1.22, 1.43]; EQ: - - Subtotal: Depression response, FU: 7-12m: RR 1.31 [1.17, 1.48]; EQ: - - Subtotal: Depression response, FU: 13-24m: RR 1.29 [1.18, 1.41]; EQ: - - Subtotal: Depression response (cluster ICC 0.00), FU: 0-6m: RR 1.32 [1.22, 1.42]; EQ: - - Subtotal: Depression response (cluster ICC 0.05), FU: 0-6m: RR 1.34 [1.23, 1.45]; EQ: - - Subtotal: Anxiety response, FU: 0-6m: RR 1.50 [1.21, 1.87]; EQ: - - Subtotal: Anxiety response, FU: 7-12m: RR 1.41 [1.18, 1.69]; EQ: - - Subtotal: Mental QoL, FU: 0-6m: SMD 0.26 [0.13, 0.38]; EQ: - - Subtotal: Mental QoL, FU: 7-12m: SMD 0.20 [0.09, 0.31]; EQ: - - Subtotal: Mental QoL, FU: 13-24m: SMD 0.25 [0.08, 0.43]; EQ: - - Subtotal: Physical QoL, FU: 13-24m: SMD 0.10 [0.02, 0.17]; EQ: - | Others  Collaborative care vs. Usual care   - Subtotal: Antidepressant medication use, FU: 0-6m: RR 1.47 [1.33, 1.63]; EQ: - - Subtotal: Antidepressant medication use, FU: 7-12m: RR 1.43 [1.26, 1.61]; EQ: - - Subtotal: Antidepressant medication use, FU: 13-24m: RR 1.22 [1.03, 1.45]; EQ: - - Subtotal: Anxiety medication use, FU: 7-12m: RR 1.17 [1.03, 1.32]; EQ: - - Patient satisfaction: SMD 0.31 [0.13, 0.49]; EQ: - - Patient satisfaction: RR 1.27 [1.18, 1.38]; EQ: - |
| 1. Barbato 2018: Couple therapy for depression. CD004188 | Couple therapy vs. No/minimal treatment   - Depressive symptoms (FU: end of treatment): SMD ‐0.95 [‐1.59, ‐0.32]; EQ: very low - Persistence of depression (FU: end of treatment): RR 0.48 [0.32, 0.70]; EQ: very low | Symptom reduction  Couple therapy vs. Individual psychotherapy   - Relationship distress (FU: end of treatment): SMD ‐0.50 [‐0.97, ‐0.02]; EQ: - - Persistence of relationship distress (FU: end of treatment): RR 0.71 [0.51, 0.98]; EQ: -   Others  Couple therapy vs. Drug therapy   - Dropouts: RR 0.31 [0.15, 0.61]; EQ: very low |
| 1. Baumeister 2011: Psychological and pharmacological interventions for depression in patients with coronary artery disease. CD008012 | Pharmacological Intervention vs. Placebo   - Subtotal: Depression score (FU: Short-term), SMD based on mean change scores: SMD -0.24 [‐0.38, ‐0.09]; EQ: - - Depression remission (FU: Short-term): OR 1.80 [1.18, 2.74]; EQ: - | Utilisation/Costs  Pharmacological Intervention vs. Placebo   - Subtotal: Resource utilization, Hospitalizations: OR 0.58 [0.39, 0.85]; EQ: - |
| 1. Churchill 2013:'Third wave' cognitive and behavioural therapies versus treatment as usual for depression. CD008705 | Third wave CBT vs. Treatment as usual   - Clinical response (FU: posttreatment): RR 0.51 [0.27, 0.95]; EQ: very low - Clinical remission (FU: posttreatment): RR 0.77 [0.67, 0.88]; EQ: very low - Depression levels (FU: posttreatment): SMD ‐1.12 [‐1.53, ‐0.71]; EQ: very low   Third wave CBT vs. Types of control condition   - Subtotal: Clinical response at posttreatment, Third wave CBT vs. Treatment as usual/usual care/no treatment: RR 0.51 [0.27, 0.95]; EQ: - |  |
| 1. Cooney 2013: Exercise for depression. CD004366 | Exercise vs Control   - Subtotal: Quality of life, Environment, SMD 0.62 [0.06, 1.18]; EQ: - - Subtotal: Quality of life, Physical, SMD 0.45 [0.06, 0.83]; EQ: - | Symptom Reduction  Exercise vs Control   - Reduction in depression symptoms (FU: posttreatment, SMD ‐0.62 [‐0.81, ‐0.42] ; EQ: moderate - Reduction in depression symptoms (FU: follow‐up), SMD ‐0.33 [‐0.63, ‐0.03] ; EQ: low |
| 1. Hunot 2013: 'Third wave' cognitive and behavioural therapies versus other psychological therapies for depression. CD008704 | Third wave CBT vs. Other psychological therapies   - Depression levels at FU: MD ‐4.51 [‐7.47, ‐1.55]; EQ: very low |  |
| 1. Ijaz 2018: Psychological therapies for treatment-resistant depression in adults. CD010558 | Psychotherapy with usual care vs. Usual care alone   - Self‐reported depressive symptoms (FU: ≤ 6m) ‐ Beck Depression Inventory (BDI): MD ‐4.07 [‐7.07, ‐1.07]; EQ: moderate - Subtotal: Self‐reported depressive symptoms (FU: ≤ 6m) – BDI, Cognitive-behavioral therapy (CBT) with usual care vs. usual care alone: MD ‐4.56 [‐7.49, ‐1.63]; EQ: - - Self‐reported depressive symptoms (FU: ≤ 6m) ‐ Patient health questionnaire (PHQ)‐9: MD ‐4.66 [‐8.72, ‐0.59]; EQ: - - Self-reported depressive symptoms (FU: ≤ 6m) ‐ SMD (BDI & PHQ‐9): SMD ‐0.40 [‐0.65, ‐0.14]; EQ: moderate - Subtotal: Self-reported depressive symptoms (FU: ≤ 6m) ‐ SMD (BDI & PHQ‐9), CBT with usual care vs. usual care alone: SMD ‐0.35 [‐0.56, ‐0.13]; EQ: - - Clinician‐rated depressive symptoms (FU: ≤ 6m) ‐ Hamilton Depression Rating Scale (HAMD): MD ‐3.28 [‐5.71, ‐0.85]; EQ: - - Response (50% reduction in depressive symptoms from baseline) (FU: ≤ 6m): RR 1.80 [1.20, 2.69]; EQ: low - Response (50% reduction in depressive symptoms from baseline) (FU: 7-12m): RR 1.73 [1.42, 2.10]; EQ: - - Remission (< 7 on HAMD or < 10 on BDI) (FU: ≤ 6m): RR 1.92 [1.46, 2.52]; EQ: moderate - Remission (< 7 on HAMD or < 10 on BDI) (FU: 7-12m): RR 1.97 [1.51, 2.56]; EQ: - |  |
| 1. Pollok 2019: Phsychological therapies for the treatment of depression in chronic obstructive pulmonary disease. CD012347 | Psychological therapies vs. No intervention   - Change in QoL (measured by St. George's Respiratory Questionnaire): MD 5.60 [0.23, 10.98]; EQ: - | Symptom reduction 31  Psychological therapies vs. No intervention   - Change in depressive symptoms: SMD 0.19 [0.05, 0.33]; EQ: - - Change in depressive symptoms (clinically depressed): SMD 0.20 [0.02, 0.37]; EQ: -   Psychological therapies versus education   - Change in depressive symptoms: SMD 0.23 [0.06, 0.41]; EQ: - |
| 1. Shinohara 2013: Behavioural therapies versus other psychological therapies for depression. CD008696 | Behavioural therapies (BT) vs. All other psychological therapies   - Subtotal: Remission, BT vs. CBT: RR 0.87 [0.75, 1.00]; EQ: low   BT vs. All other psychological therapies (FU: within 6m)   - Subtotal: Response, BT vs. CBT: RR 0.76 [0.59, 0.99]; EQ: low - Remission: BT vs. CBT: RR 0.77 [0.61, 0.98]; EQ: - - Subtotal: Depression severity, BT vs. CBT: SMD 0.42 [0.04, 0.81]; EQ: - |  |
| 1. Wilkinson 2016: Continuation and maintenance treatments for depression in older people.CD006727 | Antidepressant vs. Placebo   - Subtotal: Recurrence, FU: 12m: RR 0.67 [0.55, 0.82]; EQ: low - Subtotal: Recurrence, at final FU: RR 0.65 [0.48, 0.87]; EQ: - - Recurrence (FU: 12m, fixed-effect): RR 0.67 [0.54, 0.82]; EQ: - - Recurrence (FU: 24m): RR 0.70 [0.50, 0.99]; EQ: - |  |
| 1. Wilson 2008: Psychotherapeutic treatments for older depressed people. CD004853 | CBT vs. Control   - Reduction in symptoms (Hamilton Depression Rating Scale (HDRS)): MD ‐9.85 [‐11.97, ‐7.73]; EQ: -   Cognitive bibliotherapy vs Control   - Reduction in symptoms (HDRS): MD ‐9.29 [‐11.65, ‐6.93]; EQ: -   CBT vs. Active control   - Reduction in symptoms (HDRS): MD ‐5.69 [‐11.04, ‐0.35]; EQ: - | Others  CBT vs. Control   - Dropout (Peto): OR 0.43 [0.27, 0.68]; EQ: - |

**E-Table 9: Cancer**

| **Short Title** | **Independence and Functional Health** | **Symptom reduction, Mortality, Utilisation/Costs, Adverse Events, Others** |
| --- | --- | --- |
| 1. Bennett 2016: Educational interventions for the management of cancer‐related fatigue in adults. CD008144 | Educational interventions vs. Control (usual care or attention control)   - Anxiety: MD ‐1.47 [‐2.76, ‐0.18]; EQ: low - Global QoL: MD 11.47 [1.29, 21.65]; EQ: low | Symptom reduction  Educational interventions vs. Control (usual care or attention control)   - General fatigue: SMD ‐0.27 [‐0.51, ‐0.04]; EQ: low - Fatigue intensity: SMD ‐0.28 [‐0.51, ‐0.04]; EQ: moderate - Fatigue distress: SMD ‐0.57 [‐1.09, ‐0.05]; EQ: low   Fatigue interference: SMD ‐0.35 [‐0.54, ‐0.16]; EQ: moderate Utilization/Costs  Educational interventions vs. Control (usual care or attention control)   - Use of fatigue management strategies: SMD 0.23 [0.04, 0.41]; EQ: - |
| 1. Cavalheri 2017: Preoperative exercise training for patients with non-small cell lung cancer. CD012020 |  | Symptom reduction  Intervention group vs. Control group   - Risk of developing a postoperative pulmonary complication: RR 0.33 [0.17, 0.61]; EQ: low - Number of days patients needed an intercostal catheter: MD ‐3.33 [‐5.35, ‐1.30]; EQ: low - Preoperative exercise capacity (6-minutes walk distance (6mWD)): MD 18.23 [8.50, 27.96]; EQ: low   Forced vital capacity (% predicted): MD 2.97 [1.78, 4.16]; EQ: -  Utilisation/Costs  Intervention group vs. Control group   - Postoperative length of hospital stay: MD ‐4.24 [‐5.43, ‐3.06]; EQ: low |
| 1. Cramp 2012: Exercise for the management of cancer‐related fatigue in adults. CD006145 |  | Symptom reduction  Exercise vs. No exercise control   - Fatigue (FU: post anti‐cancer therapy): SMD ‐0.44 [‐0.79, ‐0.09]; EQ: - |
| 1. Dragan 2013: Screening for prostate cancer.CD004720 |  | Symptom reduction  Screening vs. Control   - Tumour stage (localised T1‐T2, N0, M0): RR 1.79 [1.19, 2.70]; EQ: low - Tumour stage (advanced T3‐4, N1, M1): RR 0.80 [0.73, 0.87]; EQ: moderate |
| 1. Gøtzsche 2013: Screening for breast cancer with mammography. CD001877 |  | Symptom reduction  Screening with mammography vs. No screening   - Number of cancers: RR 1.29 [1.23, 1.35]; EQ: -   Utilisation/Costs  Screening with mammography vs. No screening   - Number of mastectomies and lumpectomies: RR 1.35 [1.26, 1.44]; EQ: - - Number of mastectomies: RR 1.20 [1.11, 1.30]; EQ: - - Number treated with radiotherapy: RR 1.32 [1.16, 1.50]; EQ: - - Number treated with hormone therapy: RR 0.73 [0.55, 0.96]; EQ. –   Mortality  Screening with mammography vs. No screening   - Deaths ascribed to breast cancer (FU: 7years (y)): RR 0.81 [0.72, 0.90]; EQ: - - Subtotal: Deaths ascribed to breast cancer (FU: 7y), Suboptimally randomised trials: RR 0.71 [0.61, 0.83]; EQ: - - Deaths ascribed to breast cancer (FU: 13y): RR 0.81 [0.74, 0.87]; EQ: - - Subtotal: Deaths ascribed to breast cancer (FU: 13y), Suboptimally randomised trials: RR 0.75 [0.67, 0.83]; EQ: - - Deaths ascribed to breast cancer (FU: 7y, women ≥50y of age): RR 0.72 [0.62, 0.85]; EQ: - - Subtotal: Deaths ascribed to breast cancer (FU: 7y, women ≥50y of age), Suboptimally randomised trials: RR 0.67 [0.56, 0.81]; EQ: - - Deaths ascribed to breast cancer (FU: 13y, women <50y of age): RR 0.84 [0.73, 0.96]; EQ: - - Subtotal: Deaths ascribed to breast cancer (FU: 13y, women <50y of age), Suboptimally randomised trials: RR 0.80 [0.64, 0.98]; EQ: - - Deaths ascribed to breast cancer (FU: 13 y, women women ≥50y of age): RR 0.77 [0.69, 0.86]; EQ: - - Subtotal: Deaths ascribed to breast cancer (FU: 13 y, women women ≥50y of age), Suboptimally randomised trials: RR 0.70 [0.62, 0.80]; EQ: - - Subtotal: Mortality among breast cancer patients in the Two‐County study (FU: 7y), Mortality from cancers other than breast cancer: RR 2.42 [1.00, 5.85]; EQ: - |
| 1. Jassim 2015: Psychological interventions for women with non‐metastatic breast cancer. CD008729 | CBT vs. Control   - SMD in the change from baseline in depression: SMD ‐1.01 [‐1.83, ‐0.18]; EQ: low - SMD in the change from baseline mean change in anxiety: SMD ‐0.48 [‐0.76, ‐0.21]; EQ: low - Subtotal: SMD in the change from baseline mean change in anxiety, Group delivered intervention (<20hours (hrs)): SMD ‐0.44 [‐0.85, ‐0.03]; EQ: - | Symptom reduction  CBT vs. Control   - SMD in the change from baseline mood disturbance: SMD ‐0.28 [‐0.43, ‐0.13]; EQ: moderate - Subtotal: SMD in the change from baseline mood disturbance, Group delivered intervention (<20hrs): SMD ‐0.34 [‐0.57, ‐0.11]; EQ: - |
| 1. Ling Woo 2012: Centralisation of services for gynaecological cancer. CD007945 |  | Mortality  Institutions with gynaecologic oncologists on site (specialised centres) vs. Community or general hospital   - Ovarian cancer: survival: Hazard Ratio (HR) 0.90 [0.82, 0.99]; EQ: -   Teaching or regional cancer centre vs. Community or general hospital   - Overall survival: HR 0.91 [0.84, 0.99]; EQ: - |
| 1. Manser 2013: Screening for lung cancer. CD001991 |  | Mortality  Lung cancer screening with chest radiography +/‐ sputum cytology vs. Less intense screening   - Subtotal: Lung cancer mortality, More frequent chest x‐ray screening vs. less frequent screening: RR 1.11 [1.00, 1.23]; EQ: - - Subtotal: Lung cancer 5-year survival, More frequent chest x‐ray screening vs. less frequent: RR 0.91 [0.84, 0.99]; EQ: low |
| 1. Mishra 2012: Exercise interventions on health‐related quality of life for people with cancer during active treatment. CD008465 | Exercise interventions (varied) vs. usual care   - Overall quality of life change score, FU: ≤ 3m, SMD 0.47 [0.16, 0.79]; EQ: very low - Overall physical function change score, FU: ≤ 3m, SMD 0.69; [0.16, 1.22], EQ: very low - Overall physical function change score, FU: ≤ 6m, SMD 0.28; [-0.00, 0.55], EQ: very low - Overall role function change score, FU: ≤ 3m, SMD 0.48; [0.07, 0.90], EQ: very low - Overall social function change score, FU: ≤ 3m, SMD 0.54; [0.03, 1.05], EQ: very low | Symptom reduction  Exercise interventions (varied) vs. usual care   - Overall fatigue change score, FU: ≤ 3m, SMD ‐0.73 [‐1.14, ‐0.31]; EQ: very low |
| 1. Mustafa 2013: Psychological interventions for women with metastatic breast cancer. CD004253 | Psychological intervention vs. Usual practice   - Pain at 1y: MD ‐0.58 [‐0.98, ‐0.18]; EQ: - - Subtotal: Pain at 1y, Supportive/expressive group therapy: MD ‐0.58 [‐0.98, ‐0.18]; EQ: - | Mortality  Psychological intervention vs. Usual practice   - Survival at 1y: Odds Ratio (OR) 1.46 [1.07, 1.99]; EQ: - - Subtotal: Survival at 1y, Supportive/expressive group therapy: OR 1.56 [1.07, 2.28]; EQ: - |
| 1. Peddle 2019: Exercise training for advanced lung cancer. CD012685 * | Exercise training vs. Control   - Exercise capacity (6mWD): MD 63.33 [3.70, 122.96]; EQ: low - Disease‐specific global health‐related QoL: SMD 0.51 [0.08, 0.93]; EQ: low |  |
| 1. Poort 2017: Psychosocial interventions for fatigue during cancer treatment with palliative intent. CD012030 | Physical functioning vs. Usual care or control condition   - Physical functioning (FU: post intervention): SMD 0.32 [0.01, 0.63]; EQ: very low | Symptom reduction  Fatigue vs. Usual care or control condition   - Fatigue (First FU): SMD ‐0.66 [-1.00, ‐0.32]; EQ: - |
| 1. Rivas-Ruiz 2019: Outpatient treatment for people with cancer who develop a low-risk febrile neutropaenic event. CD009031 |  | Utilisation/Costs  Outpatients vs. Inpatients ‐ adults   - Duration of hospitalisation (days): MD ‐1.64 [‐2.22, ‐1.06]; EQ: low |
| 1. Treanor 2016: Non‐pharmacological interventions for cognitive impairment due to systemic cancer treatment. CD011325 |  | Symptom reduction  Compensatory strategy training vs. Wait‐list control immediately post‐intervention   - Psychological well‐being: SMD ‐0.57 [‐0.98, ‐0.16]; EQ: - |

**E-Table 10: COPD / asthma**

| **Short Title** | **Independence and Functional Health** | **Symptom reduction, Mortality, Utilization/Costs, Adverse Events, Others** |
| --- | --- | --- |
| 1. Freitas 2013: Breathing exercise for adults with asthma. CD001277 | Breathing exercise vs. Inactive control   - AQLQ (FU: 8w-3m): MD 0.79 [0.50, 1.08]; EQ: very low | Symptom reduction  Breathing exercise vs. Inactive control   - Asthma symptoms: MD ‐3.22 [‐6.31, ‐0.13]; EQ: very low |
| 1. Gatheral 2017: Personalised asthma action plans for adults with asthma. CD011859 | Personalised asthma action plans vs. No Personalised asthma action plans   - QoL (change from baseline in Asthma Quality of Life Questionnaire (AQLQ)): MD 0.18 [0.05, 0.30]; EQ: low |  |
| 1. Gendron 2018: Active mind-body movement therapies as an adjunct to orin comparison with pulmonary rehabilitation for people with chronic obstructive pulmonary dieseas. CD012290 * | Active mind-body movement therapy vs. Pulmonary rehabilitation   - Subtotal: SGRQ, Total: MD ‐5.83 [‐8.75, ‐2.92]; EQ: low | Active mind-body movement therapy vs. Pulmonary rehabilitation   - FEV1 predicted: MD 3.95 [1.77, 6.13]; EQ: - |
| 1. Gibson 2002a: Self-management education and regular practitioner review for adults with asthma. CD001117 | Self-management vs. Usual Care   - QoL Total Score (mean): SMD 0.29 [0.11, 0.47]; EQ: - | Symptom reduction  Self-management vs. Usual Care   - Nocturnal Asthma (% subjects): RR 0.67 [0.56, 0.79]; EQ: - - PEF (mean): SMD 0.18 [0.07, 0.29]; EQ: -   Utilisation/Costs  Self-management vs. Usual care   - Hospitalisation (% subjects hospitalised): RR 0.64 [0.50, 0.82]; EQ: - - Emergency room visits (% subjects): RR 0.82 [0.73, 0.94]; EQ: - - Emergency room visits (mean): SMD ‐0.36 [‐0.50, ‐0.21]; EQ: - - Unscheduled doctor visits (% subjects): RR 0.68 [0.56, 0.81]; EQ: - - Days off work (% subjects): RR 0.79 [0.67, 0.93]; EQ: - - Days off work (mean): SMD ‐0.18 [‐0.28, ‐0.09]; EQ: - - Total Direct Costs (mean): SMD 0.39 [0.10, 0.68]; EQ: - - Total Indirect Costs (mean): SMD ‐0.40 [‐0.69, ‐0.11]; EQ: - |
| 1. Gibson 2002b: Limited (information only) patient education programs for adults with asthma. CD001005 |  | Symptom reduction  Limited (Information only) patient education vs. Usual care   - Asthma symptoms, Peto: OR 0.44 [0.26, 0.74]; EQ: - |
| 1. Hill 2018: Neuromuscular electrostimulation for adults with chronic obstructive pulmonary disease. CD010821 | Neuromuscular electrostimulation vs. Usual care   - Peripheral muscle force: SMD 0.34 [0.02, 0.65]; EQ: low - Peripheral muscle endurance/fatigability: SMD 1.36 [0.59, 2.12]; EQ: low - Exercise capacity: 6mWD (m): MD 39.26 [16.31, 62.22]; EQ: low - Exercise capacity: endurance time (min): MD 3.62 [2.33, 4.91]; EQ: - - Symptoms: leg fatigue reported at end of exercise: MD ‐1.12 [‐1.81, ‐0.43]; EQ: -   Neuromuscular electrostimulation plus exercise vs. exercise only   - Exercise capacity: 6mWD (m): MD 25.87 [1.06, 50.69]; EQ: very low - Functional performance: days to first transfer out of bed: MD ‐4.98 [‐8.55, ‐1.41]; EQ: very low | Symptom reduction  Neuromuscular electrostimulation vs. Usual care   - Exercise capacity: VO_2_peak (L/min): MD 0.10 [0.00, 0.19]; EQ: - |
| 1. Howcroft 2016: Action plans with brief patient education for exacerbations in chronic obstructive pulmonary disease. CD005074 | Action plan vs. Usual care   - SGRQ overall score (FU: 12m): MD ‐2.82 [‐4.81, ‐0.83]; EQ: moderate | Utilisation/Costs  Action plan vs. Usual care   - At least 1 hospital admission (FU: 12m): OR 0.69 [0.49, 0.97]; EQ: moderate - At least 1 emergency department visit (FU: 12m): OR 0.55 [0.38, 0.78; EQ: - - Courses of oral corticosteroids (FU: 12m): MD 0.74 [0.12, 1.35]; EQ: moderate - Courses of antibiotics (FU: 12m): MD 2.26 [1.82, 2.70]; EQ: moderate |
| 1. Kew 2016: Home telemonitoring and remote feedback between clinic visits for asthma. CD011714 | Home telemonitoring with feedback vs. Usual monitoring   - AQLQ: MD 0.23 [0.01, 0.45]; EQ: low | Symptom reduction  Home telemonitoring with feedback vs. Usual monitoring   - Lung function (trough forced expiratory volume at 1s (FEV1)): MD 7.21 [1.52, 12.89]; EQ: moderate   Utilisation/Costs  Home telemonitoring with feedback vs. Usual monitoring   - Subtotal: Exacerbations requiring hospital admission, Adults (17-65y) FU: 7.8m: OR 0.24 [0.06, 0.94]; EQ: moderate |
| 1. Kruis 2013: Integrated disease management interventions for patients with chronic obstructive pulmonary disease. CD009437 | Integrated disease management vs. Control   - Subtotal: SGRQ: FU: Short-term 3-12m, SGRQ-Total: MD -3.71 [-5.83, -1.59]; EQ: high - Subtotal: Chronic Respiratory Questionnaire (CRQ): FU: Short-term 3-12m, CRQ-Dyspnea: MD 1.02 [0.67, 1.36]; EQ: moderate - Subtotal: Functional exercise capacity: 6mWD mean difference, 6mWD: FU: Short-term 3-12m: MD 43.86 [21.83, 65.89]; EQ: moderate - Subtotal: Functional exercise capacity: 6mWD mean difference, 6mWD: FU: Long-term >12m: MD 16.84 [3.01, 30.67]; EQ: - - Maximal exercise capacity: cycle test (W-max): MD 6.99 [2.96, 11.02]; EQ: - | Symptom reduction  Integrated disease management vs. Control   - Subtotal: CRQ: FU: Short-term 3-12m, CRQ-Fatigue: MD 0.82 [0.46, 1.17]; EQ: - - Subtotal: CRQ: FU: Short-term 3-12m, CRQ-Emotion: MD 0.61 [0.26, 0.95]; EQ: - - Subtotal: CRQ: FU: Short-term 3-12m, CRQ-Mastery: MD 0.75 [0.38, 1.12]; EQ: - - Subtotal: CRQ: FU: Long‐term >12m, CRQ-Fatigue: MD 0.45 [0.05, 0.85]; EQ: - - Subtotal: CRQ: FU: Long‐term >12m, CRQ-Emotion: MD 0.53 [0.10, 0.95]; EQ: - - Subtotal: CRQ: FU: Long‐term >12m, CRQ-Mastery: MD 0.80 [0.37, 1.23]; EQ: - - Medical Research Council dyspnea score: MD ‐0.30 [‐0.48, ‐0.11]; EQ: - - Subtotal: FEV1 (% predicted), FEV1 (% predicted; mean change) : FU Short-term: MD 2.15 [0.38, 3.91]; EQ: - |
| 1. McCabe 2017: Computer and mobile technology interventions for self-management in chronic obstructive pulmonary disease. CD011425 | Smart technology vs. Face-to-face/digital and/or written support   - Health-related QoL (Clinical COPD Questionnaire (CCQ) + St George’s Respiratory Questionnaire (SGRQ)) (FU: 4w-6m): SMD ‐0.22 [‐0.40, ‐0.03]; EQ: low - Health-related QoL (CCQ only) (FU: ≤6m): MD ‐0.28 [‐0.44, ‐0.12]; EQ: - - Daily step count (FU: 4w-4m): MD 864.06 [369.66, 1358.46]; EQ: low | Utilisation/Costs  Integrated disease management vs. Control   - Respiratory-related hospital admissions (FU: Short-term 3-12m): OR 0.68 [0.47, 0.99]; EQ: high - Hospital days per patient (all causes) (FU: Short-term 3-12m): MD ‐3.78 [‐5.90, ‐1.67]; EQ: high |
| 1. Ngai 2016: Tai Chi for chronic obstructive pulmonary disease (COPD). CD009953 | Tai Chi vs. Usual care   - 6mWT: MD 29.64 [10.52, 48.77]; EQ: very low | Symptom reduction  Tai Chi vs. Usual care   - FEV1 (L) - pulmonary function: MD 0.11 [0.02, 0.20]; EQ: moderate - Maximal Voluntary Ventilation (%): MD 6.12 [3.91, 8.33]; EQ: - |
| 1. Normansell 2017: Interventions to improve inhaler technique for people with asthma. CD012286 | Adults: enhanced education vs. Control/usual care   - Asthma control (FU: 8-12w): OR 3.18 [1.47, 6.88]; EQ: low | Others  Adults: enhanced education vs. Control/usual care   - Correct inhalter technique (FU: 2-26w): OR 5.00 [1.83, 13.65]; EQ: moderate |
| 1. Paudyal 2014: Written emotional disclosure for asthma. CD007676 | Written emotional disclosure vs. Control group   - Subtotal: Asthma control, FU: Short-term 2-3m: SMD 0.29 [0.01, 0.58]; EQ: low |  |
| 1. Peytremann-Bridevaux 2015: Chronic disease management programmes for adults with asthma. CD007988 | Chronic disease management programme vs. Usual care   - Subtotal: Asthma-specific QoL score, Randomized controlled trials (RCTs) FU: post intervention 3-12m: SMD 0.22 [0.08, 0.37]; EQ: moderate | Symptom reduction  Chronic disease management programme vs. Usual care   - Subtotal: Asthma severity score, RCTs FU: post intervention 6-12m: SMD 0.18 [0.05, 0.30]; EQ: low - Lung function (FEV1 and PEF) (FU: post intervention): SMD 0.19 [0.09, 0.30]; EQ: - - FEV1 (% predicted) (FU: post intervention): MD 2.81 [0.99, 4.64]; EQ: - - Subtotal: PEF (L/min) (FU: post intervention), RCTs: MD 33.52 [11.38, 55.65]; EQ: - - PEF (% predicted) (FU: post intervention): MD 8.68 [3.73, 13.63]; EQ: - |
| 1. Powell 2002: Options for self-management education for adults with asthma. CD004107 |  | Peak Flow Self-management vs. Regular Doctor Review   - PEF (mean): SMD 0.16 [0.01, 0.31]; EQ: - |
| 1. Sauni 2015: Remediating buildings damaged by damness and mould for preventing or reducing respiratory tract symptoms, infections and asthma. CD007897 |  | Symptom reduction  Mould remediation vs. No intervention in houses - effects in adults   - Subtotal: Asthma‐related outcomes RCT, Wheezing in last 4 weeks (FU: 12m): OR 0.64 [0.55, 0.75]; EQ: - - Subtotal: Respiratory infections RCT, Rhinitis (FU: 12m): OR 0.57 [0.49, 0.66]; EQ: - |
| 1. Silva 2013: Inspiratory muscle training for asthma. CD003792 |  | Symptom reduction  Inspiratory muscle training vs. Control   - Maximal inspiratory pressure (cmH20) - Inspiratory muscle strength: MD 13.34 [4.70, 21.98]; EQ: low |
| 1. Tapp 2007: Education interventions for adults who attend the emergency room for acute asthma. CD003000 |  | Education vs. Usual care   - Asthma symptom scores: SMD ‐0.50 [‐0.78, ‐0.22]; EQ: -   Utilisation/Costs  Education vs. Usual care   - Hospital admission/re-admission (end of FU: median: 24w, range: 4-78w): RR 0.50 [0.27, 0.91]; EQ: high - Scheduled clinic attendance: RR 1.73 [1.17, 2.56]; EQ: - |
| 1. Yang 2016: Yoga for asthma. CD010346 | Yoga vs. Usual care/sham intervention   - Change in AQLQ score: MD 0.57 [0.37, 0.77]; EQ: moderate | Symptom reduction  Yoga vs. Usual care/sham intervention   - Asthma symptoms: SMD 0.37 [0.09, 0.65]; EQ: moderate - Forced vital capacity: SMD 0.67 [0.20, 1.14]; EQ: - - Peak expiratory flow (PEF) rate: SMD 0.73 [0.36, 1.09]; EQ: -   Others  Yoga vs. Usual care/sham intervention   - Medication usage (frequency): SMD 0.69 [0.41, 0.96]; EQ: - - Medication usage (percentage of participants with decreasing dosage): RR 5.35 [1.29, 22.11]; EQ: low |
| 1. Yorke 2006: Psychological interventions for adults with asthma. CD002982 | CBT vs. Control   - Subtotal: AQLQ, Total: MD 0.71 [0.23, 1.19]; EQ - | Symptom reduction  Biofeedback therapy vs. Control   - PEF: SMD 0.66 [0.09, 1.23; EQ: -   Others  Relaxation/hypnosis/autogenic therapy vs. Control   - Decrease/discontinuation of medication: OR 4.47 [1.22, 16.44]; EQ: - |

**E-Table 11: Falls**

| **Falls** |  |  |
| --- | --- | --- |
| 1. Crotty 2010: Rehabilitation interventions for improving physical and psychosocial functioning after hip fracture in older people. CD007624 * |  | Utilisation/Costs  Specialist nurse led care vs usual care: Discharge planning and post discharge   - Poor outcome: dead or readmitted or failed to return home: RR 0.30 [0.12, 0.70]; EQ: - |
| 1. Gillespie 2012: Interventions for preventing falls in older people living in the community. CD007146 | Exercise vs. Control   - Subtotal: Rate of falls, Group exercise: multiple categories of exercise vs. Control: RR 0.71 [0.63, 0.82]; EQ: - - Subtotal: Rate of falls, Individual exercise at home: multiple categories of exercise vs. Control: RR 0.68 [0.58, 0.80]; EQ: - - Subtotal: Rate of falls, Group exercise: Tai Chi vs. Control: RR 0.72 [0.52, 1.00]; EQ: - - Subtotal: Rate of falls, Group exercise: gait, balance or functional training vs. Control: RR 0.72 [0.55, 0.94]; EQ: - - Subtotal: Number of fallers, Group exercise: multiple categories of exercise vs. Control: RR 0.85 [0.76, 0.96]; EQ: - - Subtotal: Number of fallers, Individual exercise at home: multiple categories of exercise vs. Control: RR 0.78 [0.64, 0.94]; EQ: - - Subtotal: Number of fallers, Group exercise: Tai Chi vs. Control: RR 0.71 [0.57, 0.87]; EQ: - - Number of people sustaining a fracture: RR 0.71 [0.57, 0.87]; EQ: -   Medication provision: Vitamin D (with or without calcium) vs. Control/placebo/calcium   - Subtotal: Number of fallers, Vitamin D3 (by mouth) + calcium vs. Calcium: RR 0.70 [0.53, 0.92]; EQ: -   Surgery vs. Control   - Subtotal: Rate of falls, Cardiac pacing vs. Control: RR 0.73 [0.57, 0.93]; EQ: -   Environment/assistive technology interventions: home safety vs. Control   - Rate of falls: RR 0.81 [0.68, 0.97]; EQ: - - Number of fallers: RR 0.88 [0.80, 0.96]; EQ: -   Multifactorial intervention vs. Control   - Rate of falls: RR 0.76 [0.67, 0.86]; EQ: - | Symptom reduction  Medication provision: Vitamin D analogue vs. Placebo   - Number of people developing hypercalcaemia: RR 2.49 [1.12, 5.50]; EQ: - |
| 1. Hopewell 2018: Multifactorial and multiple component interventions for preventing falls in older people living in the community. CD012221 | Multifactorial intervention vs. Usual care or attention control   - Rate of falls (falls per person years) (FU: 3-24m): RR 0.77 [0.67, 0.87]; EQ: low - Health-related QoL: endpoint score: SMD 0.19 [0.03, 0.35]; EQ: -   Multiple intervention vs. Usual care or attention control   - Rate of falls (falls per person years) (FU: 3-24m): RR 0.74 [0.60, 0.91]; EQ: moderate - Number of people sustaining one or more falls (FU: 3-18m): RR 0.82 [0.74, 0.90]; EQ: moderate - Health-related QoL: endpoint score: SMD 0.77 [0.16, 1.39]; EQ: - - Health-related QoL (mental): endpoint score: SMD 0.69 [0.26, 1.11]; EQ: - |  |
| 1. Kendrick 2014: Exercise for reducing fear of falling in older people living in the community. CD009848 | Exercise vs. Control: primary outcome - fear of falling   - Fear of falling (measured by single-item question, falls efficacy, balance confidence and concerns about falling) FU: post intervention: SMD 0.37 [0.18, 0.56]; EQ: low   Exercise vs. Control: secondary outcomes   - Occurrence of at least one fall (FU: 2-12m): RR 0.85 [0.74, 0.98]; EQ: very low - Falls rate: RR 0.68 [0.53, 0.87]; EQ: - |  |
| 1. Sherrington 2019: Exercise for preventing falls in older people living in the community. CD012424 | Exercise vs. Control   - Rate of falls - overall analysis (FU: 3-30m): RR 0.77 [0.71, 0.83]; EQ: high - Number of fallers - overall analysis (FU: 3-25m): RR 0.85 [0.81, 0.89]; EQ: high - Number of people who experienced one or more fall-related fractures - overall analysis (FU: 4-42m): RR 0.73 [0.56, 0.95]; EQ: low - Number of people who experienced one or more falls that required medical attention - overall analysis (FU: 6-24m): RR 0.61 [0.47, 0.79]; EQ: low |  |

**E-Table 12: Visus**

| **Short Title** | **Independence and Functional Health** | **Symptom reduction, Mortality, Utilisation/Costs, Adverse Events, Others** |
| --- | --- | --- |
| No CR |  |  |

**E-Table 13: Dementia**

| **Dementia** |  |  |
| --- | --- | --- |
| 1. Bahar-Fuchs 2019: Cognitive training for people with mild to moderate dementia. CD013069 | Cognitive training vs. Control   - Change in global measure of cognition (composite) (FU: immediately post intervention): SMD 0.42 [0.23, 0.61]; EQ: moderate - Change in global measure of cognition (composite), zero correlation (FU: immediately post intervention): SMD 0.24 [0.12, 0.36]; EQ: - - Change in global measure of cognition (FU: immediately post intervention): SMD 0.65 [0.26, 1.05]; EQ: low - Change in global measure of cognition, zero correlation (FU: immediately post intervention): SMD 0.27 [0.04, 0.50]; EQ: - - Change in disease progression (FU: immediately post intervention): SMD 1.07 [0.59, 1.55]; EQ: - - Change in delayed memory (FU: immediately post intervention): SMD 0.81 [0.29, 1.32]; EQ: very low - Change in immediate memory (FU: immediately post intervention): SMD 0.74 [0.37, 1.12]; EQ: - - Change in attention and working memory (FU: immediately post intervention): SMD 0.56 [0.08, 1.05]; EQ: - - Change in language (naming) (FU: immediately post intervention): SMD 0.62 [0.11, 1.12]; EQ: - - Change in verbal category fluency (FU: immediately post intervention): SMD 0.52 [0.23, 0.81]; EQ: - - Change in executive function (FU: immediately post intervention): SMD 0.75 [0.28, 1.22]; EQ: -   Cognitive training vs. Control   - Change in global measure of cognition (composite) (FU: medium-term 3-12m):SMD 0.65 [0.11, 1.20]; EQ: very low - Change in global measure of cognition (composite), zero correlation (FU: medium-term 3-12m): SMD 0.40 [0.09, 0.71]; EQ: - - Change in a global measure of cognition (FU: medium-term 3-12m): SMD 1.33 [0.31, 2.34]; EQ: very low - Change in a global measure of cognition, zero correlation (FU: medium-term 3-12m): SMD 0.68 [0.06, 1.30]; EQ: - - Change in disease progression (FU: medium-term 3-12m): SMD 0.55 [0.12, 0.98]; EQ: very low - Change in delayed memory (FU: medium-term 3-12m): SMD 0.97 [0.02, 1.92]; EQ: very low - Change in immediate memory (FU: medium-term 3-12m): SMD 0.62 [0.00, 1.24]; EQ: - - Change in language (naming) (FU: medium-term 3-12m): SMD 0.71 [0.07, 1.34]; EQ: - - Change in verbal category fluency (FU: medium-term 3-12m): SMD 0.78 [0.38, 1.18]; EQ: - - Change in executive function (FU: medium-term 3-12m): SMD 0.56 [0.02, 1.10]; EQ: -   Cognitive training vs. Alternative treatment   - Change in capacity for ADL (FU: immediately post intervention): SMD ‐0.25 [‐0.43, ‐0.07]; EQ: moderate |  |
| 1. Forbes 2015: Exercise programs for people with dementia. CD006489 | Exercise vs. Usual care: ADL   - Comparison of ADL: SMD 0.68 [0.08, 1.27]; EQ: low |  |
| 1. Gates 2019: Computerised cognitive training for preventing dementia in people with mild cognitive impairment. CD012279 | Computerised cognition-based interventions vs. Active control   - Subtotal: Global cognitive function, End of trial: SMD ‐0.53 [‐1.06, ‐0.01]; EQ: very low - Subtotal: Global cognitive function, FU: Short time point 12w-1y: SMD ‐1.23 [‐1.89, ‐0.56]; EQ: very low - Subtotal: Episodic Memory, FU: End of trial: SMD ‐0.79 [‐1.54, ‐0.04]; EQ: very low - Subtotal: Episodic memory, FU: Immediate time point 12w): SMD ‐0.99 [‐1.80, ‐0.19]; EQ: very low - Subtotal: Episodic memory, FU: Short time point 12w-1y): SMD ‐1.39 [‐2.35, ‐0.44]; EQ: very low - Subtotal: Working memory, FU: End of trial: SMD ‐0.88 [‐1.73, ‐0.03]; EQ: very low - Subtotal: Working memory, FU: Immediate time point 12w: SMD ‐0.66 [‐1.26, ‐0.06]; EQ: very low - Subtotal: Working memory, FU: Short time point 12w-1y: SMD ‐1.29 [‐1.88, ‐0.69]; EQ: very low |  |
| 1. Lins 2014: Efficacy and experiences of telephone counselling for informal carers of people with dementia. CD009126 | Telephone counselling vs. No intervention   - Depressive Symptoms: SMD 0.32 [0.01, 0.63]; EQ: moderate |  |
| 1. Liu 2018: Mindfulness-based stress reduction for family carers of people with dementia. CD012791* | Mindfulness-based stress reduction vs. Active control   - Depressive symptoms (FU: immediately post intervention): SMD ‐0.63 [‐0.98, ‐0.28]; EQ: low |  |
| 1. Smith 2015: Enhanced rehabilitation and care models for adults with dementia following hip fracture surgery. CD010569 |  | Utilisation/Costs  Interdisciplinary geriatric rehabilitation (inpatient and community rehabilitation) vs. Conventional rehabilitation   - Number of participants in institutionalised care (hospital or nursing home) (FU: 3m post-hip fracture): OR 0.46 [0.22, 0.95]; EQ: - |
| 1. Woods 2012: Cognitive stimulation to improve cognitive functioning in people with dementia. CD005562 | Cognitive stimulation vs. No cognitive stimulation   - Cognition (FU: posttreatment): SMD 0.41 [0.25, 0.57]; EQ: - - Subtotal: Cognition, Information/Orientation (FU:1-3m): SMD 0.57 [0.01, 1.14]; EQ: - - Well-being & QoL (FU: posttreatment): SMD 0.38 [0.11, 0.65]; EQ: - | Symptom reduction  Cognitive stimulation vs. No cognitive stimulation   - Communication and social interaction (FU: posttreatment): SMD 0.44 [0.17, 0.71]; EQ: - |
| 1. Woods 2018: Reminiscence therapy for dementia. CD001120 | Reminiscence therapy vs. No treatment   - Cognition (overall) (FU: posttreatment): SMD 0.11 [0.00, 0.23]; EQ: high | Symptom reduction  Reminiscence therapy vs. No treatment   - Communication and interaction (FU: posttreatment): SMD ‐0.51 [‐0.97, ‐0.05]; EQ: low - Communication and interaction (at FU): SMD ‐0.49 [‐0.77, ‐0.21]; EQ: - |

**E-Table 14: Care management, not disease specific**

| **Short Title** | **Independence and Functional Health** | **Symptom reduction, Mortality, Utilization/Costs, Adverse Events, Others** |
| --- | --- | --- |
| 1. Cochrane 2016: Time-limited home-care reablement services for maintaining and improving the functional independence of older adults. CD010825 | Reablement vs. Usual care   - Functional status (FU: 3m): SMD ‐0.40 [‐0.81, 0.00]; EQ: - - Functional status (FU: 9-12m): SMD ‐0.30 [‐0.53, ‐0.06]; EQ: very low |  |
| 1. Coulter 2015: Personalised care planning for adults with chronic or long-term health conditions. CD010523 | Personalised care planning vs. Usual care  Psychological health   - Depression (1.5-12m): SMD ‐0.36 [‐0.52, ‐0.20]; EQ: moderate | Symptom reduction  Personalised care planning vs. Usual care  Physical health   - Haemoglobin A1c (HbA1c) (change) (6-12m): MD -0.24 [-0.35, -0.14]; EQ: moderate - SBP (change) (6-12m): MD ‐2.64 [‐4.47, ‐0.82]; EQ: moderate   Personalised care planning vs. Usual care  Self-management capabilities   - Self-efficacy (1.5-12m): SMD 0.25 [0.07, 0.43]; EQ: moderate   Personalised care planning vs. Usual care  Self-care activities   - Self-care (days per week): SMD 0.35 [0.17, 0.52]; EQ: - |
| 1. Eccleston 2014: Psychological therapies (Internet-delivered) for the management of chronic pain in adults. CD010152 | Internet‐delivered psychological therapy vs. Active control, treatment‐as‐usual, or waiting‐list control  Headache   - Pain (FU: posttreatment): RR 7.28 [2.67, 19.84]; EQ: - - Disability(FU: posttreatment): SMD ‐0.65 [‐0.91, ‐0.39]; EQ: -   Non-Headache   - Pain (FU: posttreatment): SMD ‐0.37 [‐0.59, ‐0.15]; EQ: - - Disability (FU: posttreatment): SMD ‐0.50 [‐0.79, ‐0.20]; EQ: - - Depression (FU: posttreatment): SMD ‐0.19 [‐0.35, ‐0.04]; EQ: - - Anxiety (FU: posttreatment): SMD ‐0.28 [‐0.49, ‐0.06]; EQ: - - Disability (FU): SMD ‐0.15 [‐0.28, ‐0.01]; EQ: - |  |
| 1. Gomes 2013: Effectiveness and cost-effectiveness of home palliative care services for adults with advanced illness and their caregivers. CD007760 |  | Utilisation/Costs  Home palliative care vs. Usual care   - Death at home (FU: 3-24m): OR 2.21 [1.31, 3.71]; EQ: high - Death at home with only high quality RCTs (FU: 3-24m): OR 1.75 [1.24, 2.47]; EQ: high - Death in hospital: OR 0.31 [0.12, 0.79]; EQ: - |
| 1. Goncalves-Bradley 2017: Early discharge hospital at home. CD000356 |  | Utilisation/Costs  Early discharge hospital at home vs. Inpatient care for those recovering from a stroke   - Institutional care (FU: 3-6m): RR 0.63 [0.40, 0.98]; EQ: low - Hospital length of stay: MD ‐6.68 [‐10.19, ‐3.17]; EQ: moderate   Early discharge hospital at home vs. Inpatient care for older people with a mix of conditions   - Institutional care - older patients with a mix of conditions (FU: 1y): RR 0.69 [0.48, 0.99]; EQ: low - Hospital length of stay - older people with a mix of conditions: MD ‐6.76 [‐10.60, ‐2.92]; EQ: moderate - Total length of stay - older people with a mix of mainly medical conditions: MD 6.43 [2.84, 10.03]; EQ: moderate   Early discharge hospital at home vs. Inpatient care following elective surgery   - Hospital length of stay - older people recovering from surgery: MD ‐4.44 [‐6.37, ‐2.51]; EQ: moderate - Total length of stay - older people having elective surgery: MD 2.79 [0.77, 4.81]; EQ: - |
| 1. Heneghan 2016: Self-monitoring and self-management of oral anticoagulation. CD003839 |  | Symptom reduction  Self‐monitoring or self‐management vs. Standard care  Thromboembolic events   - Self-monitoring and self-management (FU: 3-57m): RR 0.58 [0.45, 0.75]; EQ: moderate - Events by Clinical Condition (FU: 3-57m): RR 0.52 [0.38, 0.71]; EQ: - - Events by self-management (Sensitivity) (FU: 3-57m): RR 0.50 [0.36, 0.69]; EQ: - - Events by specialty (FU: 3-57m): RR 0.60 [0.46, 0.78]; EQ: - |
| 1. Khalil 2017: Professional, structural and organisational interventions in primary care for reducing medication errors. CD003942 * |  | Utilisation/Costs  Organisational intervention vs. Standard care   - Number of people admitted to hospital: RR 0.92 [0.86, 0.99]; EQ: low |
| 1. Laurant 2018: Nurses as substitutes for doctors in primary care. CD001271 | Substitution of doctors with nurses for primary care vs. Routine doctor‐led primary care   - QoL: SMD 0.16 [0.00, 0.31]; EQ: low | Symptom reduction  Substitution of doctors with nurses for primary care vs. Routine doctor‐led primary care   - Systolic blood pressure: MD ‐3.73 [‐6.02, ‐1.44]; EQ: moderate - Diastolic blood pressure: MD ‐2.54 [‐4.57, ‐0.52]; EQ: moderate   Utilisation/Costs  Substitution of doctors with nurses for primary care vs. Routine doctor‐led primary care   - Length of consultation: SMD 0.38 [0.22, 0.54]; EQ: moderate - Attended return visits: RR 1.19 [1.07, 1.33]; EQ: moderate   Others  Substitution of doctors with nurses for primary care vs. Routine doctor‐led primary care   - Patient satisfaction: SMD 0.08 [0.01, 0.15]; EQ: moderate |
| 1. Légaré 2018: Interventions for increasing the use of shared decision making by healthcare professionals. CD006732 |  | Others  Interventions targeting patients vs. Usual care   - Shared decision making (patient reported outcome measure, continuous) (FU: ≤ 3y): SMD 0.32 [0.16, 0.48]; EQ: very low - Knowledge: SMD 0.38 [0.16, 0.61]; EQ: - - Knowledge (categorial): Risk Difference (RD) 0.17 [0.05, 0.29]; EQ: -   Interventions targeting healthcare professionals vs. Usual care   - Shared decision making (observer based outcome measure, continuous) (FU: post intervention ≤ 3m): SMD 0.70 [0.21, 1.19]; EQ: very low   Interventions targeting both patients and healthcare professionals vs. Usual care   - Shared decision making (observer based outcome measure, continuous) (FU: ≤ 3m): SMD 1.10 [0.42, 1.79]; EQ: very low - Knowledge: SMD 0.41 [0.28, 0.53]; EQ: - - Knowledge (categorial): RD 0.28 [0.05, 0.51]; EQ: -   Interventions targeting patients vs. Other interventions targeting patients   - Shared decision making (observer based outcome measure, continuous) (FU: post-visit): SMD 0.88 [0.39, 1.37]; EQ: very low - Match between preferred and actual level of participation in decision making: RD ‐0.10 [‐0.16, ‐0.05]; EQ: - |
| 1. Pitt 2013: Consumer-providers of care for adult clients of statutory mental health services. CD004807 |  | Utilisation/Costs  Consumer-provider vs. Professional staff  Crisis/emergency service: SMD ‐0.34 [‐0.60, ‐0.07]; EQ: |
| 1. Posadzki 2016: Automated telephone communication systems for preventive healthcare and management of long-term conditions. CD009921 |  | Symptom reduction  Automated telephone communication systems vs. Usual care for managing diabetes mellitus   - Glycated haemoglobin (FU: 6m): MD ‐0.26 [‐0.50, ‐0.01]; EQ: low - Self-monitoring of diabetic foot (FU: 12m): SMD 0.24 [0.06, 0.42]; EQ: moderate   Automated telephone communication systems vs. Usual primary care and education or usual care for managing hypertension   - Systolic blood pressure (FU: 6w): MD ‐1.89 [‐2.12, ‐1.66]; EQ: moderate   Utilisation/Costs  Automated telephone communication systems vs. Control for improving health services uptake (screening rates)   - Subtotal: Breast cancer screening, Multimodal/complex interventions (FU: 12m): RR: 2.17 [1.55, 3.04]; EQ: high - Subtotal: Colorectal cancer screening, Multimodal/complex interventions (FU: 12m): RR 2.19 [1.88, 2.55]; EQ: high - Subtotal: Colorectal cancer screening, IVR (6m): RR 1.36 [1.25, 1.48]; EQ: moderate   Others  Automated telephone communication systems vs. Control for improving health services uptake (immunisation)   - Immunisation in children (FU: 4m): RR 1.25 [1.18, 1.32]; EQ: moderate - Immunisation in adolescents (FU: 15m): RR 1.06 [1.02, 1.11]; EQ: moderate |
| 1. Rosendal 2013: Enhanced care by generalists for functional somatic symptoms and disorders in primary care. CD008142 |  | Others  Enhanced care vs. Treatment as usual   - Discontinuation at FU: 6-24m: RR 1.25 [1.08, 1.46]; EQ: - - Discontinuation at FU: 1-3m: RR 1.28 [1.06, 1.54]; EQ: - |
| 1. Shepperd 2016: Hospital at home: home-based end-of-life- care. CD009231 |  | Utilisation/Costs  Place of death   - Dying at home (FU: 6-24m): RR 1.33 [1.14, 1.55]; EQ: high |
